# Influence of Process Parameters on the Characteristics of Additively Manufactured Parts Made from Advanced Biopolymers

**DOI:** 10.3390/polym15030716

**Published:** 2023-01-31

**Authors:** Tomaž Pepelnjak, Josip Stojšić, Luka Sevšek, Dejan Movrin, Mladomir Milutinović

**Affiliations:** 1 Faculty of Mechanical Engineering, University of Ljubljana, Aškerčeva 6, 1000 Ljubljana, Slovenia; 2 Mechanical Engineering Faculty in Slavonski Brod, University of Slavonski Brod, Trg Ivane Brlić Mažuranić 2, 35000 Slavonski Brod, Croatia; 3 Department for Production Engineering, Faculty of Technical Sciences, University of Novi Sad, Trg Dositeja Obradovića 6, 21000 Novi Sad, Serbia

**Keywords:** additive manufacturing, biopolymers, medical applications, process parameters, part complexity

## Abstract

Over the past few decades, additive manufacturing (AM) has become a reliable tool for prototyping and low-volume production. In recent years, the market share of such products has increased rapidly as these manufacturing concepts allow for greater part complexity compared to conventional manufacturing technologies. Furthermore, as recyclability and biocompatibility have become more important in material selection, biopolymers have also become widely used in AM. This article provides an overview of AM with advanced biopolymers in fields from medicine to food packaging. Various AM technologies are presented, focusing on the biopolymers used, selected part fabrication strategies, and influential parameters of the technologies presented. It should be emphasized that inkjet bioprinting, stereolithography, selective laser sintering, fused deposition modeling, extrusion-based bioprinting, and scaffold-free printing are the most commonly used AM technologies for the production of parts from advanced biopolymers. Achievable part complexity will be discussed with emphasis on manufacturable features, layer thickness, production accuracy, materials applied, and part strength in correlation with key AM technologies and their parameters crucial for producing representative examples, anatomical models, specialized medical instruments, medical implants, time-dependent prosthetic features, etc. Future trends of advanced biopolymers focused on establishing target-time-dependent part properties through 4D additive manufacturing are also discussed.

## 1. Introduction

**Figure 1:**
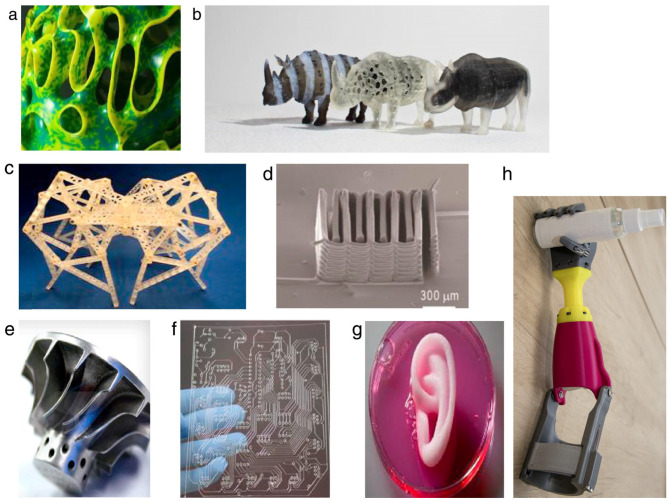
Examples of AM-produced parts: (**a**) artistic shapes inspired by nature, (**b**) three rhinos, printed using OpenFab, (**c**) Theo Jansen locomotive mechanism, (**d**) 3D printable lithium-ion rechargeable battery, (**e**) metallic turbine, (**f**) printed circuit board, (**g**) artificial printed ear [2], (**h**) printed prosthetic (courtesy Faculty of Technical Sciences Novi Sad) [5].

Current industrial practice requires manufacturers to increase the complexity of parts while reducing production and delivery times. In addition, the complexity of parts designed and produced is constantly increasing, forcing manufacturers to use advanced production processes and methods. While most parts had been produced using conventional technologies such as cutting, forming, and casting, today, for many parts, it is far from sufficient to use only these technologies. Based on the requirements of rapid part development and part presentation in the prototyping phase, the first additive technologies were reported as early as the mid- to late-1980s, while the fundamentals of additive manufacturing (AM) for various technologies were established in the 1990s. However, the first available solutions of devices and/or machines for prototyping parts were not commercially available until the 2000s. Nowadays, there are more than 50 different additive manufacturing technologies worldwide [1]. From this range, some technologies have become widely used due to their flexibility, technological advancement, applicability, or materials used. The wide variety of manufactured parts can be seen in Figure 1. It should be noted that AM technologies have also drastically changed the design principles and complexity of parts, as shown by Gao et al. [2].

The American Society for Testing and Materials (ASTM) has classified AM into seven groups in the standard ISO/ASTM 52900:2021 [3]: Jetting, Binder Jetting, Vat Photopolymerization, Powder Bed Fusion, Material Extrusion, Energy Deposition, and Film Lamination, which were evaluated by Varma et al. [4] for 3D printed scaffolds for biomedical applications. 

**Figure 2:**
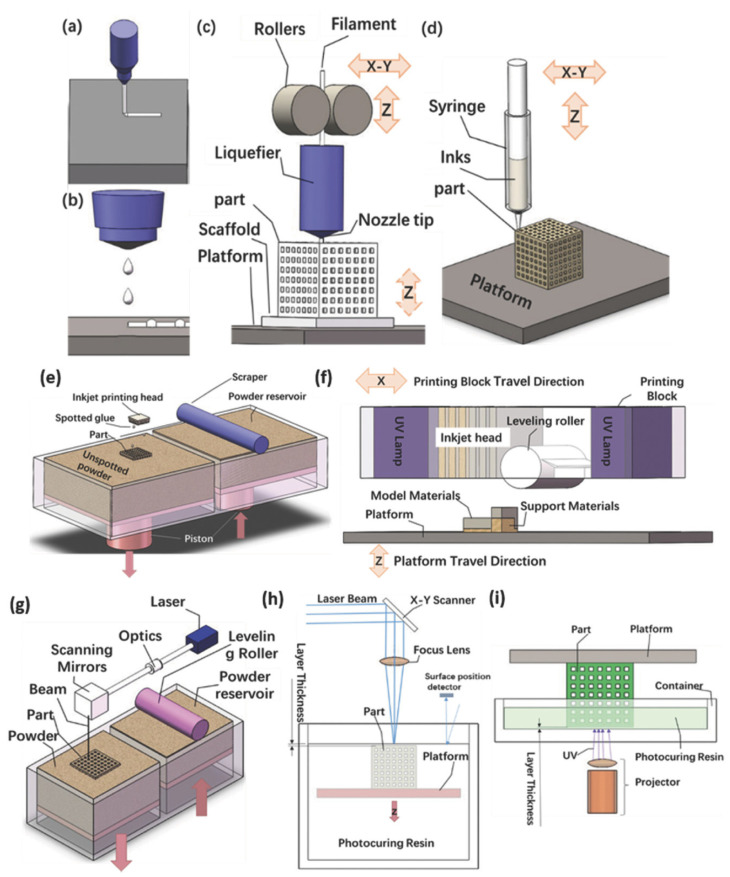
Principles of AM methods: (**a**) continuous filament writing, (**b**) droplet jetting, (**c**) fused deposition modeling, (**d**) inkjet printing, (**e**) the powder bed inkjet 3D printing, (**f**) multijet 3D printing, (**g**) selective laser sintering, (**h**) free-surface stereolithography, and (**i**) constrained-surface stereolithography [6].

The main AM concepts used today in different industries [2], from aerospace to automotive, electronics, apparel, and medical, were presented by Zhakeyev [6] as shown in Figure 2. Some individual AM technologies have shown tremendous potential, while others developed in the early years of AM have found few applications due to the unfavorable combination of the setup concept and the materials used, such as laminated object manufacturing (LOM).

To assess the scientific impact of particular technologies, as well as AM as an interruptive technology in general, their appearance in the Web of Science database was monitored. The two matching keywords (“additive manufacturing” and “3D printing”) are frequently used and were therefore evaluated together. The Boolean operator “or” was used to count the results. The matched results from the Web of Science database (WOS) provided 69,341 results, including 50,831 journal articles, 12,597 conference papers, and 800 book chapters, indicating an overall enormous research potential in this area since 1995, when Dickens [7] published his paper on research development in rapid prototyping. It should not be forgotten that the first works on rapid prototyping were written even earlier [8,9,10] and that during the same period several patents [11,12,13,14] addressed conceptual issues of rapid prototyping techniques that were not yet mature for actual AM applications. The number of scientific works from the field of AM research dealing with a particular commonly used technology was also analyzed. In this case, the Web of Science database was considered by applying the keyword of a specific technology, such as “stereolithography” and its abbreviation “SLA”, but without the keywords “AM” or “3D printing”, to obtain the total publication volume of all papers dealing with AM (see Table 1). In the table, the keywords representing the sophomores are listed together. It should also be noted that a large number of results can be obtained in the case of laser-engineered net shaping (LENS) if the process abbreviation is also inserted, since the search engine does not distinguish between LENS as a technology and “lens” as an optical element. Due to this fact, the number of hits obtained must be carefully examined.

Combining the above keywords, “AM” and “3D printing”, with the use of polymers, we find 9264 hits in WOS, of which 7739 were published in the previous five years. Focusing further on the field of biopolymers, using the keywords “bio-polymer” and “biopolymer”, WOS yields 91 results, most of which relate to various applications in the medical field. 

**Figure 3:**
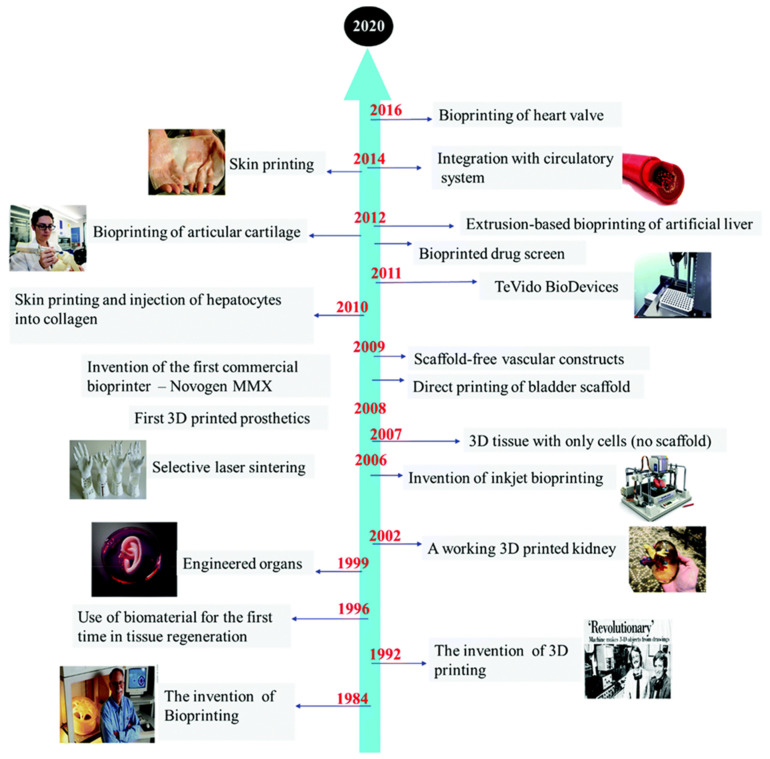
Timeline with the milestones of the bioprinting through the early beginning up to the printing of the heart valves [22].

The development of AM technologies has been influenced by several factors, ranging from basic knowledge of the physics and chemistry of the process itself to strategies for fabricating layered components, to the applicability of a particular build-up process for various research, prototyping, art [15], fashion [16], and industrial products. In recent years, even food processing by 3D printing is reported by Lee [17] and Agunbiade [18]. From the beginning, AM technologies were dedicated to rapid product development, enabling the delivery of initial parts in drastically less time than competing conventional technologies at the time. Since the early stages of AM were not capable of producing functional parts, this changed dramatically in the 2000s. Constantly improving technologies have reached a level where AM machines can produce new AM machines, duplicate parts, or produce spare parts for various industrial machines [19] to replace their worn parts. Thanks to precise equipment, microparts or parts with microfeatures can also be produced [20,21]. In particular, in medicine and biomaterial applications, AM opened several completely new application areas, for which Taghizadeh et al. [22] presented the milestones of the technologies (Figure 3). 

**Figure 4:**
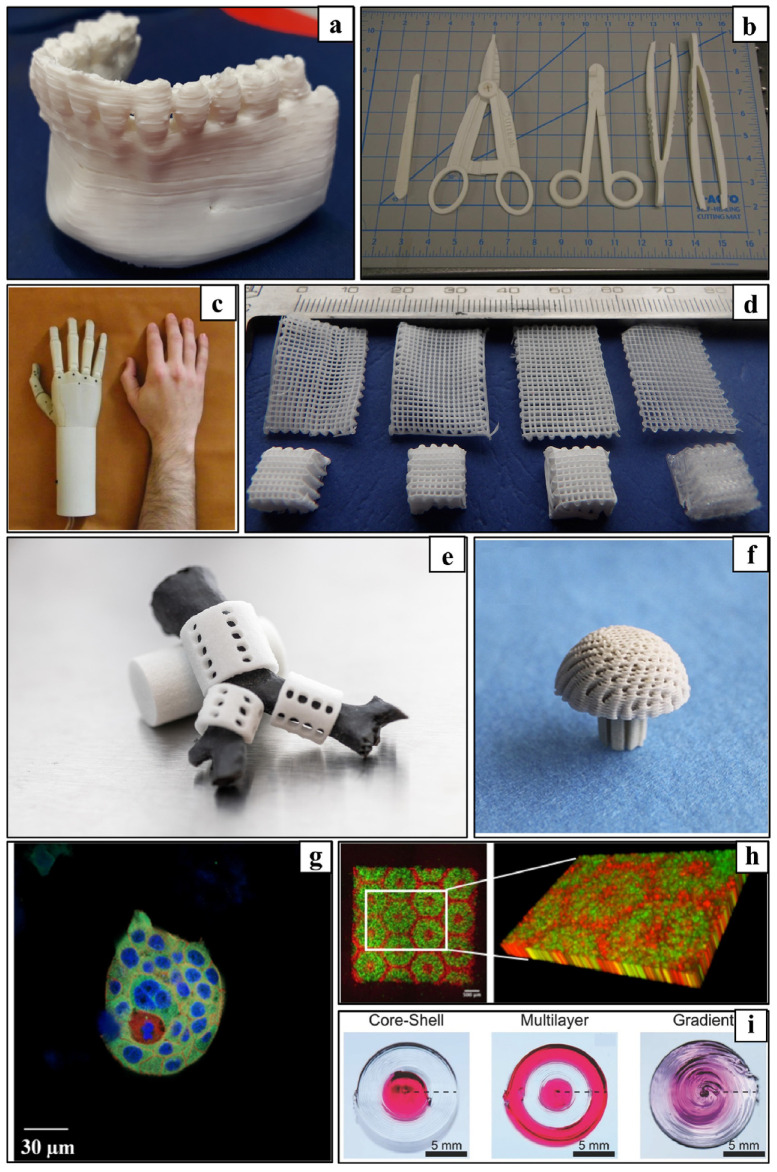
Representative examples of biomedical AM applications: (**a**) 3D anatomical models, (**b**) specially designed surgical instruments, (**c**) exo-prostheses, (**d**) 3D printed surgical implants, (**e**) surgical implants made of biodegradable materials, (**f**) scaffold-based tissue engineering, (**g**) 3D-microstructured tissue models for visualization, (**h**) advanced organ-on-a-chip integration, (**i**) time-dependent drug release system [24].

These applications can be divided into AM of prosthetic parts and dental prostheses, tissue engineering (TE), scaffolds [23], advanced medical carriers, etc., for which biodegradable biopolymers are indispensable in several cases [24] (Figure 4). The advanced materials for polymer-based scaffolds range from 100% biodegradable materials based on cellulose and other natural polymers [25] to ceramic-reinforced materials, which Cometa et al., have analyzed using BioCell Printing as an example for producing specific ceramic-reinforced scaffolds [26]. 

**Figure 5:**
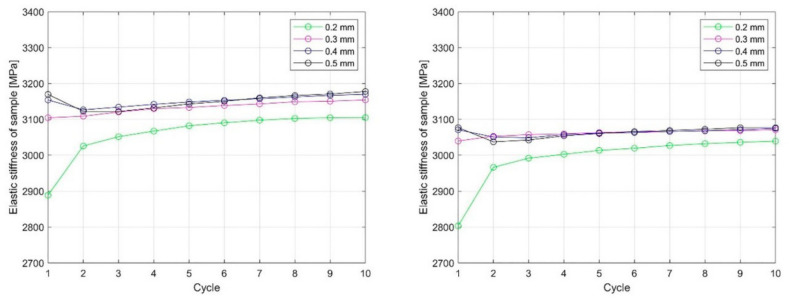
Influence of cyclic plastic loading on modulus of elasticity [34].

The selected materials [27,28,29], the additive manufacturing technology used [30], the process parameters of the selected AM [31,32] to achieve the proper mechanical properties, the manufacturing accuracy to minimize warpage during cooling of the manufactured part [33], and the complexity of the designed part all affect the usability of the manufactured part in its life cycle. There is even the possibility of selectively changing the properties of the part during its use [34], as illustrated by Pepelnjak et al., who evaluated the changes in elastic modulus due to plastic deformation of FFF-printed polylactic acid (PLA) (Figure 5), for which the circular specimens of 10 mm height were compressed by 0.2 to 0.5 mm. 

The changes in AM-printed parts must be evaluated during the design phase of the part [35,36]. Since these are the most important influencing parameters of the part, they will be described in more detail in the following sections.

## 2. Biopolymer Materials

Biopolymers can be obtained from animal, plant, and algal sources, by fermentation of microorganisms, or by enzymatic processes [37]. The term “biopolymers” refers to polymers that exhibit high biocompatibility and biodegradability, whether they are natural or synthetic [38]. Biopolymers are viable alternatives to petroleum-based plastics because they are abundant in nature and are both renewable and biodegradable [39]. The goal is to replace as many synthetic polymers as possible with biopolymers. An example of this is the study by Zorko et al. [40], in which fossil-based polyoxymethylene (POM) and polyamide 66 (PA66) were replaced in a polymer gearbox with a bio-based PA6.10.

Biopolymers can be classified into polysaccharides and proteins based on their chemical composition [37]. Some examples of polysaccharides are cellulose, gellan gum, and pectin, and some examples of proteins are collagen, gelatin, silk, and keratin [37]. Synthetic polymers have poor biocompatibility and cell adhesion, are mechanically unstable, and produce harmful by-products during the degradation process, so natural polymers are often a better choice for 3D printing requirements [22]. Some common biopolymers have poor properties or disadvantages that can affect the manufacturing process with these polymers or even the quality of the product. Such disadvantages include the shape inconsistency of collagen or the high viscosity of agarose [22]. With this in mind, we can still choose numerous biopolymers for additive manufacturing technologies, such as gelatin, silk, collagen, alginate, and their functionalized types, among others [22]. PLA is also a biopolymer with excellent mechanical properties, thermal stability, processability, and recyclability [41] and low environmental impact, and it is widely used for the fused deposition modeling (FDM) process of 3D printing, in which the raw material is used in the form of filaments [42,43]

Silk, collagen, and keratin are biopolymers and, as such, are renewable. They are derived from natural sources such as plants, skin, arthropod exoskeletons, silk cocoons, spider webs, and hair. Cellulose, in contrast, is a carbohydrate usually derived from cotton or wood and is widely used in the pharmaceutical sector [39]. Developments in processing technology and cellulose chemistry have meant that extrusion-assisted additive manufacturing of cellulose is no longer limited to traditional applications such as paper and wood products but can also be printed using various extrusion-assisted additive manufacturing processes [38]. Cellulose can also be used for various additive extrusion technologies such as melt extrusion and solution extrusion [24]. Gelatin is a natural, biocompatible, and biodegradable polymer derived from collagen, and it can also be used for the production of hydrogels that have high water content and are biocompatible [23,44]. Gelatin is also cheap and non-immune-compatible [45]. Some applications of gelatin include hydrogel formulation and controlled drug release boats, and even gelatin prototypes using hepatocyte extrusion have been printed [45]. Gelatin-based bio-inks can be used for controlled 3D printing of microarchitectures [23]. Gelatin can also be used in many additive manufacturing processes, such as solution extrusion additive manufacturing [24].

Natural biopolymers such as cellulose, chitosan, and starch are in demand for the production of collagen for tissue engineering applications [38]. Extrusion-based additive technology may be the most suitable for these types of materials because the localized power of lasers can affect the chemical properties of the aforementioned materials [38]. Chitosan is characterized by its biodegradability and biocompatibility and has also been investigated as a hydrogel for wound dressings and skin tissue development [46]. For collagen, solution extrusion and binder jet techniques, among others, can be used [24]. Collagen exhibits good biocompatibility and low antigenicity and can be tailored to optimize its mechanical properties, degradation, and water absorption [44]. Other biopolymers, such as chitosan, silk fibroin, polyhydroxyalkanoate (PHA), hyaluronic acid, and alginate, can also be used for additive manufacturing by solution extrusion [24]. In contrast, chitosan and hyaluronic acid can also be used for stereolithography (SLA) and fibrin for bioprinting [24].

**Figure 6:**
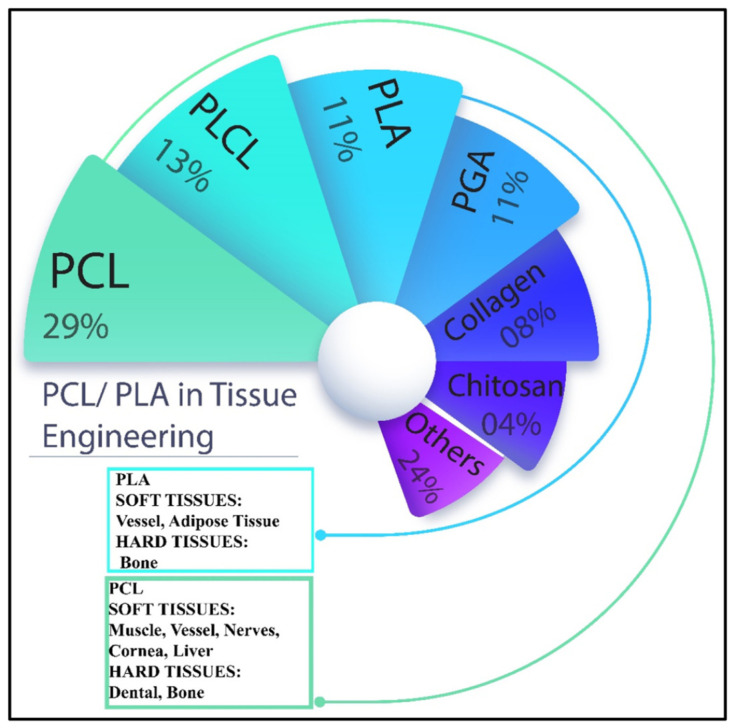
List of biopolymers used in fabrication of scaffolds for tissue regeneration [47].

Biopolymer composites are degradable in natural environments when exposed to ultraviolet (UV) light, microorganisms, or moisture [47]. Biopolymers and their composites are widely used in many biological or chemical fields, among which we can highlight the pharmaceutical or medical applications, such as the fabrication of scaffolds for tissue regeneration, for which polylactic acid (PLA), polyglycolic acid (PGA), poly(L-lactide-co-e-caprolactone) (PLCL), and even polycaprolactone (PCL) are used as examples of biocompatible and biodegradable biopolymers [45,47]. Different biopolymers used to fabricate scaffolds for tissue regeneration are shown in Figure 6. Some synthetic biopolymers, including PLA, PCL, PGA, polyethylene glycol (PEG), and poly(lactide-co-glycolide) (PLGA), have been approved by the US Food and Drug Administration (FDA) for use in biomedicine [47].

**Figure 7:**
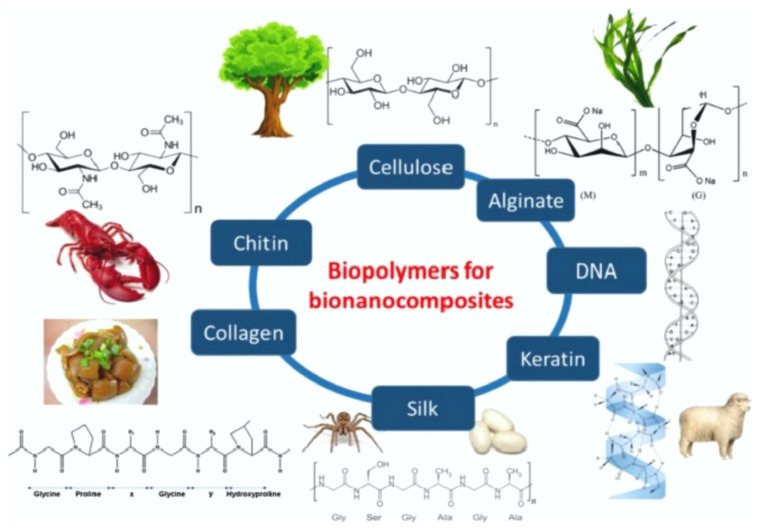
Different biopolymers that allow the development of bio-nanocomposites [39].

Nanocomposites exhibit higher mechanical strength, greater heat resistance, and even self-healing behavior compared to pure polymers and conventional composites [39]. Renewable nanoparticles, such as cellulose whiskers, polyethylene (PE), polypropylene (PP), and even epoxide, are used in bio-nanocomposites, along with many biopolymers, such as PLA and PHA [39]. Conductive fillers in a wide range of polymers with different properties, including biopolymers, can also be used in a variety of additive manufacturing processes [48]. Figure 7 shows different possible biopolymers that can be used in the development of bio-nanocomposites.

Bio-inks are a combination of various synthetic polymers and biopolymers together with a wide range of microcarriers and nanoparticles [22]. Bio-inks should be made from a suitable selection of biopolymers that can meet the rheological requirements for 3D bioprinting [22]. An example of a biopolymer is chitosan, from which suitable bio-ink can be prepared with some modifications [22]. Chitosan-based bio-inks, which are commonly used for the 3D printing of scaffolds, are biodegradable and affordable inks with a high degree of printability, cytocompatibility, biocompatibility, and mechanical stability [22]. A study by Lee at al. [49] presents the design of scaffolds coated with gold nanoparticles grown on the biopolymer polydopamine (PDA) on a 3D printed scaffold of biodegradable PCL. This development could enable progress in the field of bone tissue engineering [49]. SLA 3D printing requires photocurable liquid resins that are also biocompatible [50]. In a study by Miao et al. [50], a novel renewable epoxidized soybean oil epoxidized acrylate was solidified into biocompatible scaffolds that support the growth of multipotent human bone marrow mesenchymal stem cells. Polymers derived from vegetable oil are economical and renewable, compared to petroleum-based polymers derived from a limited resource [50].

Another group of materials is those that, when exposed to a stimulus, undergo a temporary change in shape, which may mean a return to the original shape or the retention of an unbalanced shape. These types of materials are referred to as shape-memory materials (SMMs), which include shape-memory polymers (SMPs). The vast majority of SMP materials are biocompatible, i.e., harmless to living tissues, or even biodegradable, non-toxic, and non-mutagenic [51,52,53,54]. Some SMP materials can also be activated by an external heat source or embedded rigid heating elements, which is the basis for the 4D printing paradigm in the study by Zhang et al. [55]. The 4D printing technology uses SMP polymers in the biomedical field. A variety of printing technologies can be used, including SLA and FDM [56]. In the work of Grigsby et al. [57], two biopolymers, keratin and lignin, were used with the aim of producing 4D functional materials. Grigsby et al. [57] also demonstrated that the printed keratin–lignin biocomposite material responded to moisture stimuli, thus achieving a four-dimensional response following the 3D printing FDM process. In the work of Kirillova et al. [58], hollow self-folding tubes with high control over their dimensions and geometry were fabricated using advanced 4D printing of shape-changing biopolymer hydrogels. A tube diameter of 0.02 mm was achieved, which is comparable to the diameters of the smallest blood vessels [58].

As evident from the literature review in the presented section, the analyzed materials can be divided into natural polymers and synthetic biopolymers produced mainly from natural ones. The implementation of the synthetic modification of natural polymers is dedicated to attaining the material properties necessary for the successful processing of AM, such as proper viscosity, melting temperature, and, in the case of stereolithography, photocurable biocompatible liquid resins with their mechanical and rheological properties. The materials are mainly focused on particular AM technologies for various biodegradable materials presented in the next section. As evident from Figure 2, the material’s viscosity is crucial in the case of continuous filament writing and droplet jetting while the melting temperature is crucial in all technologies for which the material needs to be melted during the AM process. In particular, the melting temperature is important in all cases for which the melt and degradation temperatures of the used biopolymer are close together. In contrast, all AM technologies applying the curing of the material or its melting by laser beam or UV beam require the exact energy intake per material volume not to exceed the material degradation during the processing by AM.

## 3. Additive Manufacturing Technologies

**Figure 8:**
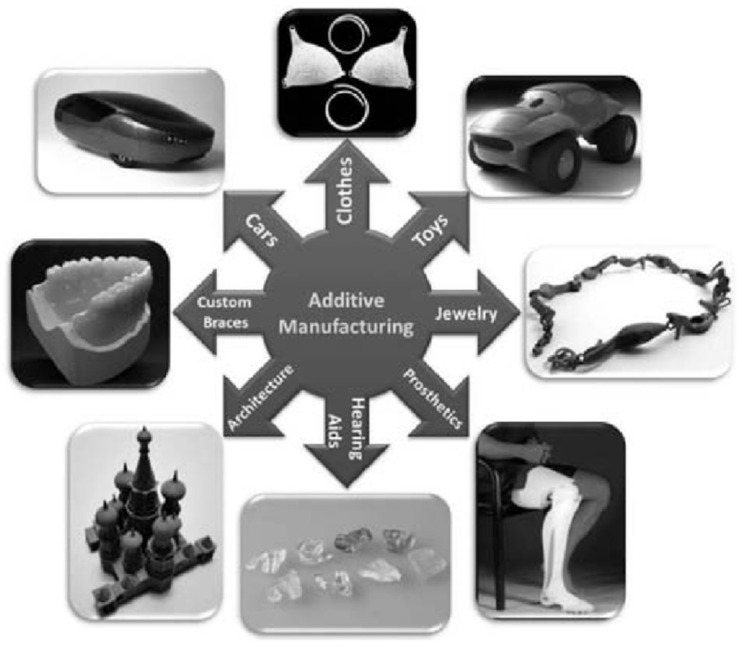
The use of AM in different industry sectors [51].

Additive manufacturing (AM) technologies offer significant advantages over other technologies, including the fact that AM technologies are competitive with injection molding up to 1000 units [51,59]. Unlike injection molding, which requires expensive tooling, 3D printing allows for low fixed costs since no special tooling is required [59]. In addition, it should be noted that 3D printing produces less material waste than material removal technologies, such as milling [59]. AM technologies also allow easier modification of the product shape according to the customer’s requirements [51]. Pieces produced using AM methods meet the criteria of the industries found in Figure 8.

Over the years, numerous 3D printing technologies have been developed. Biomaterials that can be used in extrusion-based additive manufacturing technologies include bio-ceramics, bio-metals, and biopolymers or their biocomposites [38]. Biopolymers are more readily manufactured than bio-ceramics or even bio-metallic materials due to their lower melting points and greater latitude for tempering their chemical or molecular structures by modifying their crosslinking mechanisms [60].

Bioprinting technologies can be divided into binder and material jetting, vat polymerization, powder bed fusion, and finally material extrusion [61]. In binder and material jetting bioprinting, liquid droplets of a specific printed material are applied using inkjet techniques [61]. Powder bed fusion uses a heat source, such as a laser or electron beam, to heat a metal or plastic powder bed locally, fusing it layer by layer until the final 3D product is formed [61]. Selective laser sintering (SLS) is a good example of a powder bed fusion technology that is also used in the field of tissue engineering. In material extrusion technologies, the printed material is passed through a nozzle and applied to the surface of the previously printed material layer. Two important material extrusion processes are FDM, in which the raw material is usually in the form of filaments, and extrusion-based bioprinting (EBB) [43,61]. One of the most widespread and simplest AM technologies is the extrusion of material through a moving nozzle, which allows the material to be directed to the desired locations of the platform on which the product is formed layer by layer [62]. One of the possible variants of material extrusion is the use of flowable slurries as a base material and is carried out without heating [51]. This method has been used in food extrusion and in printing biomaterials filled with living organisms, including cells, among other applications [51,63]. This 3D printing technique can also be used to print collagen to create different shapes of the base surface on which cells can grow [63]. There are many options for drug delivery, but implantable drug delivery has many advantages, including the delivery of lower doses of drugs [64]. Stewart et al. [64] focused on the use of 3D printing as a manufacturing process for implantable drug delivery devices with hot melt extrusion to produce the PLA filament, which was used in combination with polyvinyl alcohol (PVA) filament for implant manufacturing.

**Figure 9:**
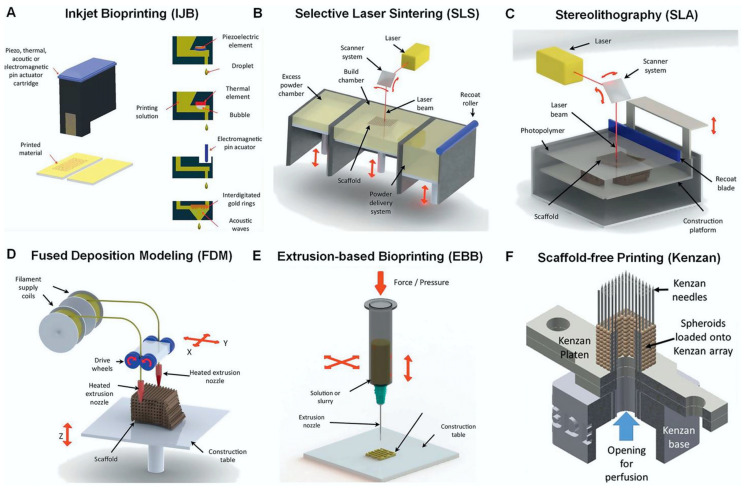
Commonly used bioprinting technologies [61].

Vat polymerization uses a photocurable liquid polymer that is selectively polymerized on the surface of the vat using a weak light source [61,65]. Two common vat photopolymerization techniques used are stereolithography (SLA) and two-photon polymerization (2PP), which are also used for scaffold fabrication [61]. Huang et al. [66] presented the stereolithography process with thermal assistance. Polyethylene glycol diacrylate (PEGDA) with the dissolved charge of the biopolymer PEG has been used for potential biomedical applications because the materials used have excellent mechanical properties, such as hardness, modulus of elasticity, and compression modulus [66]. Recently, researchers have also applied bioprinting technologies to cartilage tissue engineering, as conventional fabrication technologies cannot fully reproduce these heterogeneous and anisotropic tissues [61]. In the field of tissue engineering, a so-called scaffold-free approach that uses the inertial capabilities of the cell to fuse and produce an extracellular cartilage matrix that generates new living tissue can be mentioned [61,67]. In the so-called Kenzan process, a scaffold-free printing technique, cellular spheroids are precisely placed one by one in a microneedle array using a robotic arm [61,68]. Figure 9 shows the most commonly used bioprinting techniques.

In many biological and chemical fields, microfabrication techniques offer an advantage and an opportunity. One possible fabrication technique is photolithography, in which a substrate is hardened by optical or UV light at specific locations [69]. In contrast, soft lithography enables microstructuring using elastomeric molds, stamps, and photomasks without using the photolithographic technique [69]. Since 3D printing techniques are already found in many fields of medicine, many biopolymers have been used for various 3D printing techniques that achieve sufficient printing resolution. For example, to print a structure similar to natural bone, which consists of complex microstructures, advanced 3D printing techniques and suitable biomaterials must be used [70]. In many cases, 3D printing techniques can also be performed using biopolymers. Direct 3D ink writing allows the extrusion of inks based on prefabricated molds, also using biopolymers, such as chitosan, collagen, and alginate. Powder printing has also been used to make bone implants; in this process, the final product is made in layers, and liquid binders are added to the powder bed for each layer. In this case, powder printing can use many composite materials that also contain biopolymers such as alginate and collagen. Extrusion-based bioprinting of bone implants, which involves crosslinking of extruded bioprints composed of cells and biomolecules, can also use biopolymers, such as alginate and PEG, a hydrogel-based biopolymer [70]. Bone implants can also be fabricated using SLA for PEG biopolymer or SLS for PVA biopolymer.

Numerous additive technologies have been used in the medical and biomedical fields. One such specific area is the fabrication of biopolymer scaffolds, which, when porous, have numerous advantages for biomedical applications, particularly tissue engineering [47]. As an example, SLA is a UV light-based 3D printing technology that uses a photocurable resin that is exposed to UV light, which cures the resin. Since UV light can cause skin cancer, visible light can be used for many medical applications in SLA bioprinting. SLA can be used to synthesize biopolymers and other biopolymer-based composite scaffolds [47,71]. The digital light processing (DLP) 3D printing technology uses a projection of UV or visible light to project the shape of a layer or designed pattern. DLP allows higher printing speed with lower accuracy compared to SLA printing. DLP uses numerous biopolymers to develop biopolymer composites for tissue engineering applications [47,72]. SLS uses high laser density, which in scaffold fabrication means fusing ceramics, metals, or even polymers or composites to develop a 3D structure of the final product with excellent mechanical properties and surface quality [47]. SLS also produces highly porous products that can be used for tissue engineering applications [47,72]. FDM uses an extrusion device that provides temperature control and deposits the biopolymer onto the surface layer by layer and is also used for tissue engineering applications, but mostly in the context of post-processing strategies, as biopolymers are generally not produced with FDM due to their high melting temperature [47,73,74]. 

**Figure 10:**
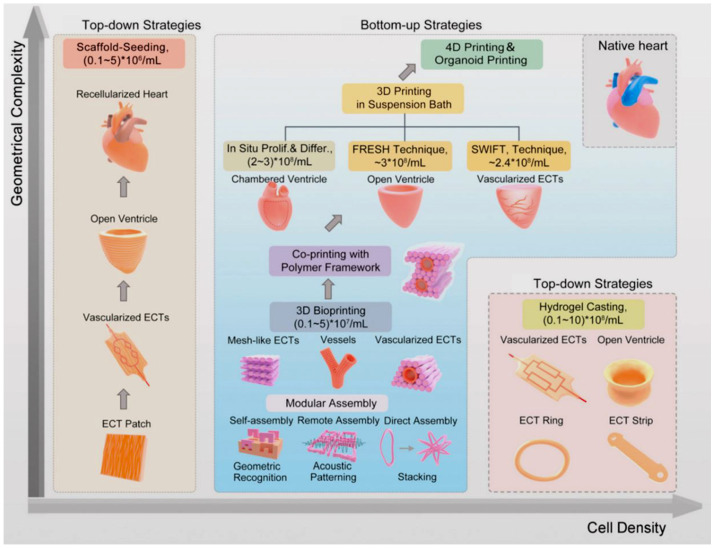
Bioengineering approaches for in vitro generation of cardiac tissue in terms of their ability to achieve the geometrical complexity and physiological cell density of the native heart [75].

Various extrusion, droplet or inkjet, and laser 3D bioprinting technologies have been used to fabricate scaffolds for tissues [47]. To improve the quality of the fabricated scaffolds, PCL- or PLA-based polymers can be combined with other natural polymers to produce hybrid scaffolds. Modification of PCL and PLA with additives, hydrogels, or even other biopolymers can improve the mechanical and even biological properties of these biomaterials [47]. In recent years, significant progress has been made in the maturation strategies for microscale cardiac tissue [75]. With the advances of new biomaterials and 3D bioprinting technologies and the new research in numerous fields of tissue engineering, countless new applications in the field of cardiac pumps or even cardiac engineering can be developed [75]. In Figure 10, bioengineering approaches for in vitro generation of cardiac tissue are presented in terms of their ability to match the geometric complexity and physiological cell density of the native heart.

Other 3D printing technologies that can also be used to fabricate biodegradable polymer-based scaffolds include extrusion-based printing, vat photopolymerization, binder jetting, and powder bed fusion [46]. A widely used tactic for obtaining biomimetic scaffolds is the use of biomaterials that have an identical composition to the tissue to be replaced [4]. The natural healing mechanism allows the damaged tissue to recover its normal structure and function, but this process is slow and not feasible for all tissues [76]. This problem is the main focus of tissue engineering, which aims to replace damaged tissue with new tissue [76]. One of the technologies that can enable the development of that field is 4D printing.

**Figure 11:**
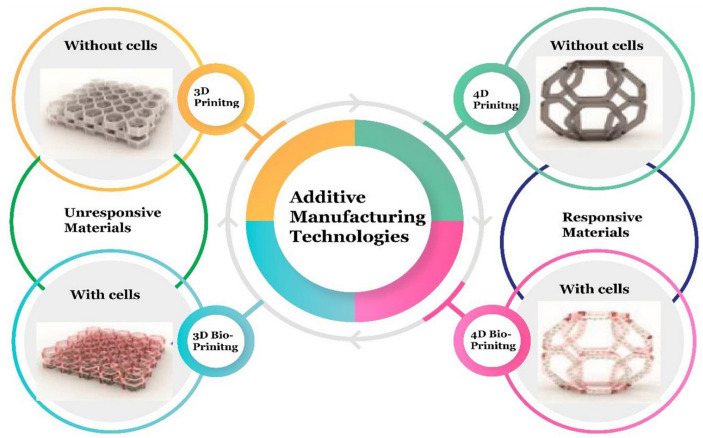
Conceptual difference between 3D and 4D printing [78].

Four-dimensional printing is a novel technology that combines AM methods with time. The general definition of this process is the specific modification of 3D printed structures in terms of shape, properties, or even functionality [51,77]. The difference between the unresponsive 3D printed and responsive 4D printed materials was presented by Arif et al. [78] (Figure 11).

Another requirement for 4D printing is the need for a stimulus that induces morphological changes. These stimuli include heat, light, water, and magnetic fields, alone or in combination [51,78,79,80,81,82,83]. Thus, the shape of a printed product can be changed by applying an external stimulus. Four-dimensional bioprinting can also be used in tissue engineering, robotics, biosensing, and drug delivery [84]. With the initial printing and subsequent modification of the properties of the printed part, 4D printing opens the way for the development of smart devices in numerous industries, including aerospace, medicine, biology, and many fields of engineering [85]. The main drawback of this technique could be the limited number of stimulus-responsive materials and suitable 4D modeling or design [85].

**Figure 12:**
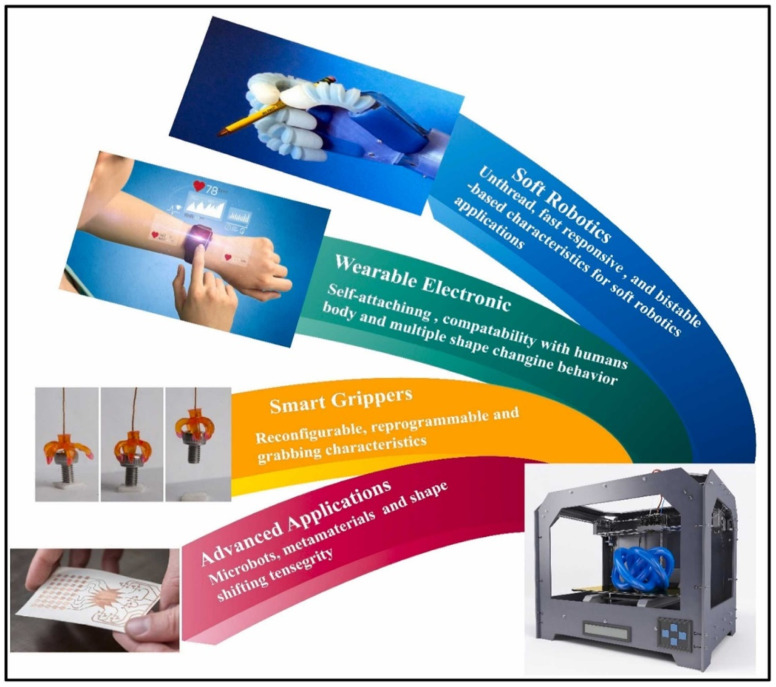
Applications of 4D printed smart materials [87].

In addition, 4D printing requires smart materials that can change in the presence of a stimulus. The change in such a material can be stretching, bending, or deformation in the presence of a stimulus [51]. The coordination of micro- and nanoprinting technologies at the human biological level enables breakthroughs in the development and engineering of artificial organs, tissues, cells, and subcellular structures [86]. This presents new challenges in the implementation of 3D and 4D printing production and development [86]. Among others, the self-bending of a 3D-bioprinting structure has been achieved, the shape change of which is caused by dissolving the corresponding material in a suitable solvent [77]. Hence, 4D printing is a further development of 3D printing, in which the fourth dimension is the time-dependent shape or other property of the product after the printing is completed [78]. The 4D printing of smart materials is thus a promising manufacturing process that can be used to produce complex structures for many fields, including biomedicine, food industry, electronics, textiles, and agriculture [87]. Figure 12 shows the 4D printing of smart materials used to develop soft devices for a wide range of engineering applications.

**Figure 13:**
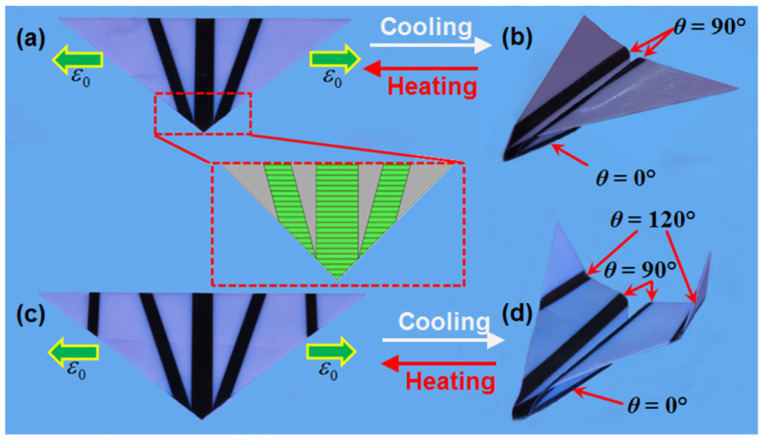
Origami airplanes produced by active 4D printing concept [78]. A flat triangle polymer sheet with three hinges in (**a**) assembles itself into an origami airplane with a 0° angle in the middle hinge that bends upward having 90° angles in the two side hinges being bend downward in (**b**). Furthermore, a flat triangle sheet with five hinges in (**c**) assembles itself into an origami airplane with two winglets as presented in (**d**).

Ge et al. [78] introduced the concept of printing an active origami structure using active composites with time-memory polymer fibers printed into an elastomer matrix, automatically folding a flat surface into a complex 3D component (Figure 13). Wan et al. [88] presented the 4D printing process of several biocompatible and biodegradable SMP polymer materials and their nanocomposites that respond to thermal and magnetic stimuli.

Another subcategory of 4D printing is 4D bioprinting, which allows the fabrication of flexible and dynamic structures of soft and hard tissues [78]. Four-dimensional bioprinting also uses materials that respond to stimuli, including SMP polymers, as well as crosslinked structures made of different or the same types of polymers, which in some cases are biopolymer hydrogels [78]. Four-dimensional bioprinting compounds, also referred to as vigorous origami or shaping systems, use similar 3D printing methods to create structures by layering biopolymers [56]. As a result of the use of various stimuli-responsive biomaterials and a range of 4D bioprinting techniques, these 4D bioprinted structures can undergo morphological or functional changes over time [56]. Four-dimensional bioprinting technology can also be used for tissue regeneration, addressing unmet medical needs [56].

It is evident that the analyzed AM technologies and concepts are strongly product-oriented. Therefore, the appropriate selection of the material combined with the right choice of the optimal AM technology for a newly designed part has a crucial role.

## 4. Manufacturing Parameters for AM of Advanced Biopolymers

The design and development of AM processes differ greatly from those of traditional manufacturing methods. In general, the process design for additive manufacturing can be divided into six (main) steps: part/model orientation, 3D model slicing, process variable selection and optimization, support generation, path generation, and post-processing determination [89,90]. As with traditional manufacturing technologies, process design and process parameters have a dominant impact on both AM product attributes (cost, part accuracy, surface quality, mechanical and physical properties, etc.) and AM process efficiency. Selecting and optimizing AM process parameters to meet product and process requirements is a highly challenging task since there are seven distinct AM process categories with many process inputs and process variables that could be varied in a wide range. In addition, the part geometry and the material play an important role in setting optimal process parameters for the majority of AM technologies, making the task even more demanding [89]. It should be noted that the base of AM-compatible materials is growing continuously but this material development is not always accompanied by the AM process parameter development, which in turn is a critical issue for introducing new materials [91]. The main design aspects and manufacturing parameters for the most commonly employed AM technologies in processing biopolymer materials will be discussed in the continuation of this review.

### 4.1. Vat-Based Polymerization Processes

**Figure 14:**
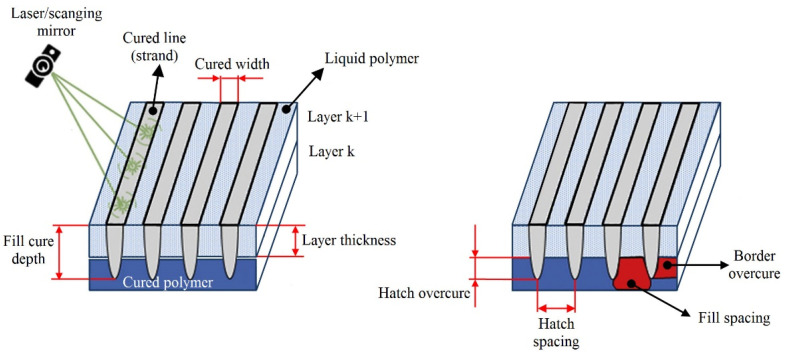
Build parameters of the SLA process [100].

Extensive studies [92,93,94,95,96,97,98,99,100] have shown that vat polymerization processes (SLA, DLP) are affected by various process parameters. According to Schaub et al. [92], there are over fifty process variables that cause errors and affect the physical and mechanical properties of SLA parts. Different criteria may be used to classify SLA parameters. Zakeri et al. [101] divided them into two groups: technical parameters, which can be adjusted using the SLA system, and photosensitive parameters, which are the intrinsic properties of the resin. The technical parameters can be further classified into three categories: part/build parameters, support parameters, and recoat parameters, with build parameters being the most important because of their decisive influence on part quality and SLA process efficiency [93]. Figure 14 displays a schematic representation of the build parameters, which include layer thickness, hatch spacing, fill spacing, border overcure, hatch overcure, and fill cure depth [93,94]. Another important build parameter is part orientation, which influences not only the component accuracy and surface quality but also the need for supporting structures, part strength, hardness, part build time, and, as a result, the part cost [102,103].

**Figure 15:**
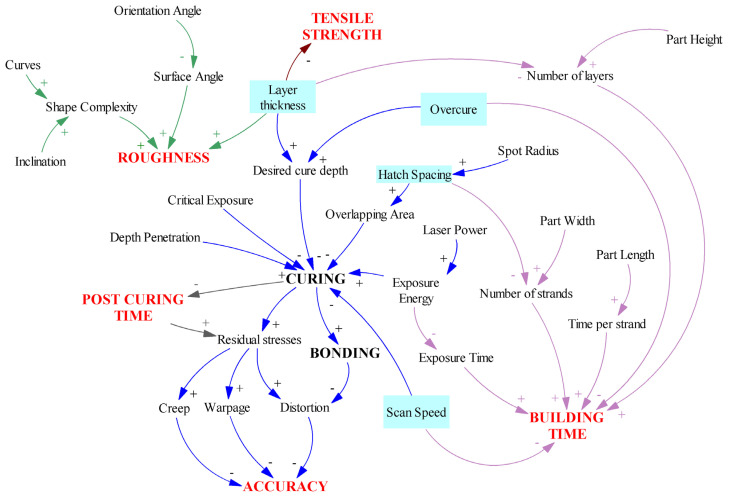
Casual loop diagrams for SLA process parameters [102].

Planar dimensions (horizontal resolution) depend greatly on the quality/characteristics of a machine laser system (laser power and resolution, stability of the laser beam, etc.), but, in general, these are not adjustable process parameters. However, it is possible to control laser-scanning velocity and path (trajectory). Both parameters affect the quality and efficiency of the SLA process; increasing these parameters reduces the processing time but causes lower quality [97]. The vertical resolution is governed by the layer thickness and the depth of light penetration, which may be varied/controlled by the type and amount of absorbers added to the photopolymer resin. Presented in Figure 15 is a casual loop diagram, which illustrates the relationships and interaction between the SLA process parameters, and their effect on the mechanical properties (tensile strength) and quality (the dimensional accuracy and surface roughness) of SLA-printed parts as well as the efficiency of the SLA process (building time, post-curing time). In this diagram, positive links (+) indicate that the increase in a cause (certain process parameter) will result in an increase in the effect. In contrast, negative links (−) indicate that if the cause increases, the effect decreases, and if the cause decreases, the effect increases. 

**Figure 16:**
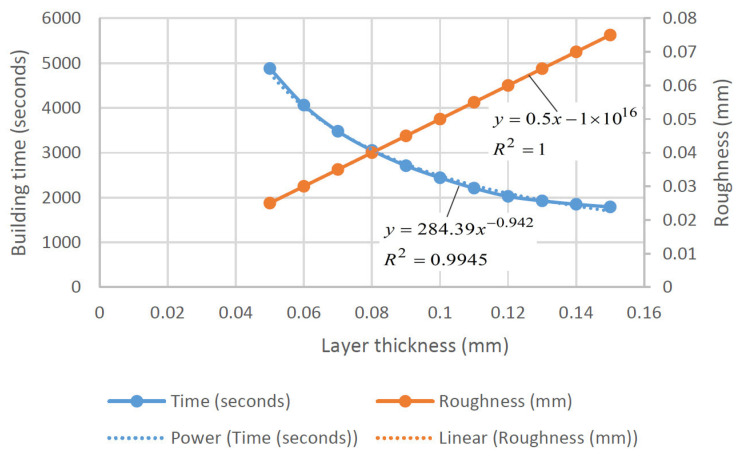
The effect of layer thickness on building time and roughness [102].

It can be seen from Figure 15 that the five marked process parameters (layer thickness, overcure, spot radius/hatch spacing, and scan speed) have different effects on the observed features. For example, overcuring has differing effects on two independent responses (accuracy and building time). A small amount of overcuring leads to better part curing, stronger bonds, and less residual stresses. This result is desirable in terms of part dimensional accuracy, but it will increase the part building time. Spot radius, which directly influences hatch spacing and overlapping area, exhibits the same (conflicting) effect on building time and accuracy. By using a large spot radius, the building time will be reduced, but poor bonding will occur due to large un-cured regions between parallel hatch lines, which leads to part distortion and poor accuracy. Similarly, a small layer thickness provides a good surface finish and tensile strength of parts; it also enhances curing and bonding and minimizes residual stresses, resulting in better accuracy. However, the number of layers needed to build the part increases, as does the building time. El-Sherif [102] found that the relationship between layer thickness and roughness is linear, while layer thickness and building time are related by the power trend line equation (Figure 16). The power trend line was also observed between laser power and exposure time, laser power and building time, and laser spot size and building time. A second-order polynomial best approximates the change in roughness as a function of surface angle. 

The design principles of the SLA process for bio-based polymers are essentially the same as for all other materials. Certainly, the most challenging task when developing the SLA process for biopolymers is the formulation of the photocuring resin/system. This primarily refers to biopolymers used in the field of biomedicine for drug delivery, hard and soft tissue engineering, etc., for which photosensitive resin must fulfill rigorous standards in terms of both the functional (biocompatibility, biodegradability, and bioactivity) and the structural (transparency, elasticity, strength, etc.) requirements. According to Ferreira et al. [104], the biocompatibility of the photo-initiator is a critical issue to be considered when biomedical applications are concerned. Several photo-initiators have been developed to address a range of photopolymerization applications, but the majority of them are cytotoxic (cause cell death) and do not meet critical biocompatibility criteria [105,106].

Significant progress was achieved with the introduction of Irgacure 2959, which, together with lithium phenyl-2,4,6-trimethylbenzoylphosphinate (LAP) and eosin Y, is the most commonly used photo-initiator for biomedical applications [107]. According to the mechanism of free radical generation, Irgacure-2959 and LAP are photo-cleavable (type I) photo-initiators, whereas eosin Yi is a biomolecular (type II) photo-initiator. Lists of photo-reactive biopolymers and their photo-initiators can be found elsewhere [105,108,109]. In addition to the biocompatibility standards, total or partial solubility in water [108] and absorption spectra in the UV A range (315–400 nm) or visible light (400–700 nm) are other basic requirements of a photo-initiator used for biomedical applications [110]. The quantity of photo-initiator in the photopolymer resin is also a significant parameter; by varying this parameter, the absorption spectra of photo-initiators may be changed. Visible light-sensitive photo-initiators are less cytotoxic and more water-soluble than UV light-sensitive photo-initiators, but they cure at a slower rate due to the lower energy level of visible light [111]. Arifin et al. [112] provided a short review of the effects of various SLA process parameters, such as curing time, power light source/intensity, resolution, layer thickness, and scan velocity on the cured thickness (fill cure depth) of solidified resin, mechanical properties, biocompatibility, and porosity of a biomedical component (i.e., a TE scaffold). It was found that the influence of the process parameters relies not only on the process parameter setup but also on the type and viscosity of the resin, meaning that scaffold properties are defined by the combination of all of these factors. For example, the curing depth, which is one of the most difficult parameters to control in the SLA process, is affected by the exposure dosage (light intensity and illumination time/scanning speed) as well as the resin employed. For biomedical applications, the resin typically polymerizes under mild conditions: low light intensity (energy), short irradiation time, physiological temperature, and low organic solvent levels [113]. Reduced light intensity and heating effect result in reduced shrinkage and part distortion, which positively affect the accuracy of biomedical components. 

### 4.2. Fused Deposition Modeling (FDM)

FDM technology is one of the most complex technologies, given the number of possible parameter combinations directly responsible for final part properties. The main parameters influencing the mechanical, dimensional, and morphological characteristics of the parts are layer thickness, printing temperature parameters (head and bed temperature), speeds (print and non-print moves), infill (shape and percent), and others [114,115,116,117,118]. Various permanent or temporary implants, drug delivery systems, and medical equipment can be produced using FDM technology [119,120,121].

**Figure 17:**
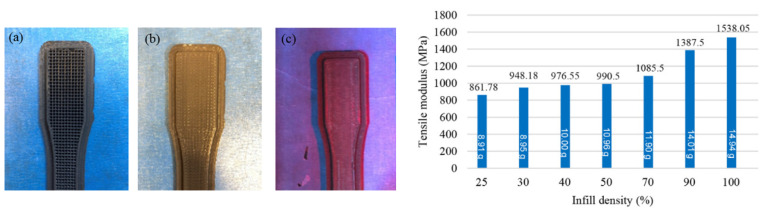
FDM printed specimens with different infill densities: (**a**) 25% (**b**) 50%, (**c**) 100%; the relationship between the tensile modulus and the infill density for pure PLA [125].

The most popular material used in FDM technology is PLA [122], the main characteristics of which are biodegradability, biocompatibility, non-toxicity, non-immunogenicity, non-inflammatory properties, and printability [123], which makes it one of the best materials in the biomedical field. PLA is also suitable as a base for composite material for FDM technology, where it can be combined with different materials and elements, such as magnesium, copper, silver, and others. In research studies [114,115,124], PLA material is used in combination with nanoparticles to make composites to provide different levels of biodegradability and higher mechanical properties of printed scaffolds. The mechanical properties of printed PLA parts can be maximized using optimal process parameters. In the specimen or model preparation phase, software for FDM allows variation of part/model density using additional infill percent, which significantly influences mechanical properties, such as tensile strength. Abeykon et al. [125] experimentally proved that by increasing infill from 25 to 100%, the tensile strength almost doubled (Figure 17), and the weight of specimens increased from 9 to 15 g. This ratio (density/weight–mechanical properties) is significant when a load-bearing bone reconstruction is necessary. Similar research was provided by Hikmat et al. [126], who used the Taguchi method to determine the influence of seven printing parameters on the mechanical properties of printed specimens and suggested and confirmed optimal parameter values. According to the research, only three parameters are statistically significant: build orientation, nozzle diameter, and infill density. The morphology of the PLA depends on the process heat parameters (head and bed temperature) and plays a significant role in the biomedical field because, in some cases, morphological changes can cause cytotoxicity. For example, the percentage of PLA crystallinity greatly influences cell adhesion and viability in tissue engineering applications, which is explained by Sarasua et al. [127]. Different cells responded in a specific manner depending on the composition and the crystalline characteristics of the substrates.

Today, one of the most popular materials for clinical implantation is polyether ether ketone (PEEK). It has better mechanical properties and thermal stability than PLA, excellent chemical resistance, and a perfect combination of strength, toughness, and stiffness. Because of the high thermal stability of printed PEEK implants, they need devices that can achieve a very high temperature of the printing head (up to 520 °C) and the bed (up to 160 °C) [128]. The temperature of the printing head and bed of the printer not only affects the mechanical properties of PEEK-printed parts but also the relative part density [129]. The relative density of PEEK increases gradually with the nozzle temperature, which indicates that the air pores are partially decreasing due to better material fluidity. During the printing of PEEK polymer, it is necessary to control not only head and bed temperature but also ambient temperature, because the thermal stress effect is much more significant than that in the PLA case and must be managed [130]. Aside from standard process parameters, the orientation of parts, relative to the printer coordinate system, can influence mechanical properties, surface roughness, and morphological changes, (e.g., the level of crystallinity) [131]. In the same study, the authors optimized the dimensional accuracy of the printed parts using five process parameters: layer thickness, infill percent, number of shells, and type of infill pattern. Choosing the right combination of parameters, in this case, the layer thickness of 0.15 mm, infill percentage of 80%, two shells, and a rectilinear infill pattern, the changes in dimensions were reduced by only 0.232 mm. Dimensional accuracy is significant, but it does not need to be high for all clinical applications, which allows a wider range of values of printing parameters. Layer thickness and raster angle significantly affect tensile, compressive, and maximal bending stress and must be chosen correctly. According to Wu et al. [132], the optimal mechanical properties of PEEK will be achieved at a layer thickness of 0.3 mm and a raster angle of 0°/90°.

Table 2 summarizes the influences of main printing parameters on three printing properties, mechanical properties, surface quality, and morphological changes. With a plus sign, a strong influence on printing properties is marked, while a minus sign means a minor influence.

### 4.3. Selective Laser Sintering

**Figure 18:**
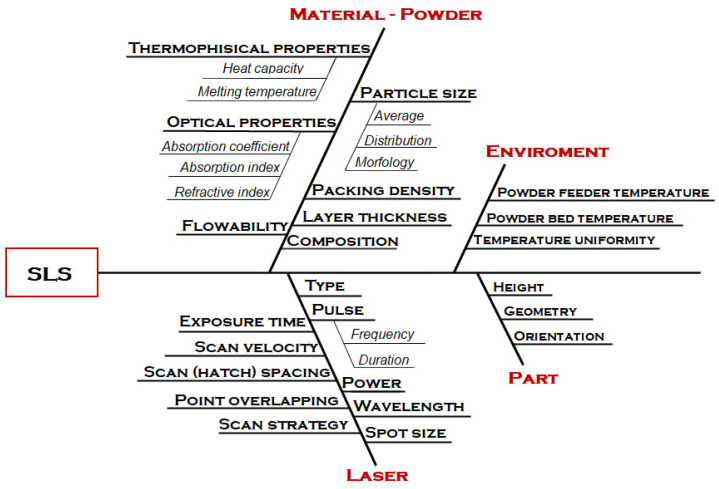
Ishikawa diagram of SLS process parameters.

As in SLA and FDM, the structural and mechanical properties of SLS-printed components are affected by several process parameters. Han et al. [133] classified all SLS process parameters into two groups: laser parameters and build parameters. In contrast, Riza et al. [134] divided the parameters into four basic categories: laser-based, temperature-based, scan-based, and powder-based. Figure 18 shows the Ishikawa or fishbone diagram illustrating the main variables influencing the SLS process. 

Laser power (*P_L_*), scan speed (*v_s_*), and scan spacing (hatch distance *H*) are recognized as the most critical SLS process parameters [134,135]. These parameters determine the energy density (*E_A_*) on the powder surface (Equation (1)), which is a key factor in terms of the mechanical properties, quality, and accuracy of sintered parts. The energy density is also known as the Andrew number, which is a quantitative measure of laser input power and laser scan parameters. This factor is independent of the material processed, and hence, it could help in standardizing the interpretation of results between different SLA printers.

(1) $$E_{A}=\frac{P_{L}}{v_{s}\cdot H}$$

To account for the impact of layer thickness, the surface-related energy density (*E_A_*) can be extended to a volume-related energy density (*E_V_*) with layer thickness (*d_l_*) as a variable, as given in Equation (2)

(2) $$E_{V}=\frac{P_{L}}{v_{s}\cdot H\cdot d_{l}}$$

According to Equations (1) and (2), a higher energy density is obtained by setting a higher laser power and lesser scanning speed, hatch distance, and layer thickness. The increase in energy density is accompanied by an increase in mechanical properties and part density, but only to a certain degree. Further rise in the energy density may cause the material to degrade, resulting in a drop in mechanical properties and dimensional accuracy. The energy density, particularly scanning speed, has a significant impact on powder particle fusion, and hence on part density/porosity. Lower scanning speeds enhance the energy density and contact time between the laser beam and the particles, causing the melting process to be more intense. This increases the amount of liquid phase available to fill the voids between the particles, which promotes sintering and leads to a denser structure [134]. Therefore, a balance between laser power and scan speed is required. In addition to energy density, the quality of the laser sintering process and the SLS part strength are determined by the energy absorption, which is a function of the laser beam wavelength and SLS material used. The most commonly utilized lasers in SLS systems for polymer processing are CO_2_ lasers with wavelengths of 10.6 μm [134,136]. Studies on the absorbance of various materials at different SLS laser wavelengths have revealed that polymer absorbance improves at high wavelengths (infrared domain) [137]. When designing the SLS process, special attention should be paid to the scan path. This variable most significantly affects manufacturing time and material shrinkage, i.e., the dimensional accuracy of SLS components [138]. In general, as the scan length increases, the shrinkage ratio increases, which has a negative impact on accuracy. In addition, the scan path determines the part’s density and, as a result, its mechanical characteristics. Manufacturing time and shrinkage ratio are also closely related to the layer thickness. According to Dadbakhsh et al. [139], the shrinkage ratio decreases rapidly with increasing layer thickness but only up to a certain value of thickness (0.2 mm). Further layer thickness increases have no effect on the relationship between layer thickness and shrinkage ratio. When considering manufacturing time, increasing the layer thickness could reduce it, but this results in an increase in surface roughness and poor resolution of printed parts, which can be improved by later post-processing [140]. In terms of SLS printing resolution, other influencing factors include laser spot size and powder particle characteristics, such as particle size distribution and geometry [141]. Typical SLS powders with good flowabilities and density characteristics are composed primarily of spherulite particles with a narrow size distribution of *d* = 60 μm and with a low share of fine particles smaller than *d* = 10 μm [142]. Powders with uniform particle size distribution and high density improve the print resolution, porosity, mechanical properties, and accuracy of SLS parts. Finer powder (powder with a smaller average size of particles) allows a much higher processing temperature and packing density compared to coarse powder [141]. At higher temperatures, the maximum density of the part is achieved at lower energy density, which significantly reduces thermal stresses and material degradation.

**Figure 19:**
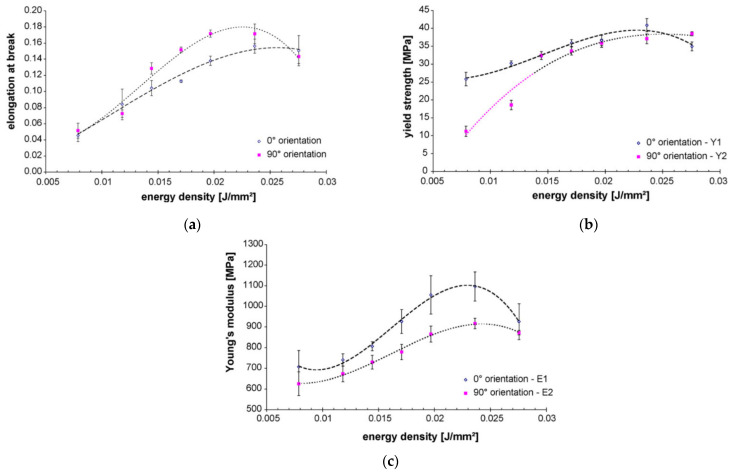
Influence of energy density on (**a**) elongation at break, (**b**) yield strength, and (**c**) Young’s modulus of SLS-sintered PA specimens [146].

Numerous studies have examined the influence of energy density and other SLS process parameters on the characteristics of SLS components fabricated from biopolymers [143,144,145,146]. Caulfield et al. [146] studied the effect of energy density (*E_A_*) on the physical and mechanical parameters of sintered PA specimens. The results of the research showed that material properties, such as elongation at break, yield strength, Young’s modulus, fracture strength, and relative density exhibit strong dependence on the *E_A_* level (Figure 19); all the parameters increase with an increase in *E_A_* level, except for the case of the highest *E_A_* level (over 0.025 J/mm^2^). Increasing *E_A_* levels were also seen to result in larger part dimensions. As for the recommended minimum value of the energy density, PA objects should not be built below 0.012 J/mm^2^ unless a porous structure is necessary. For processing biopolymer PEEK 450 PF, the optimal energy density in terms of mechanical properties and part quality is very similar (i.e., 0.029 J/mm^2^) [147]. It should be noted that in this case, a further increase in the energy density led to a slight increase in the tensile strength; however, the results of the ANOVA analysis revealed that this increase was not statistically significant as the risk of material degradation increased. 

**Figure 20:**
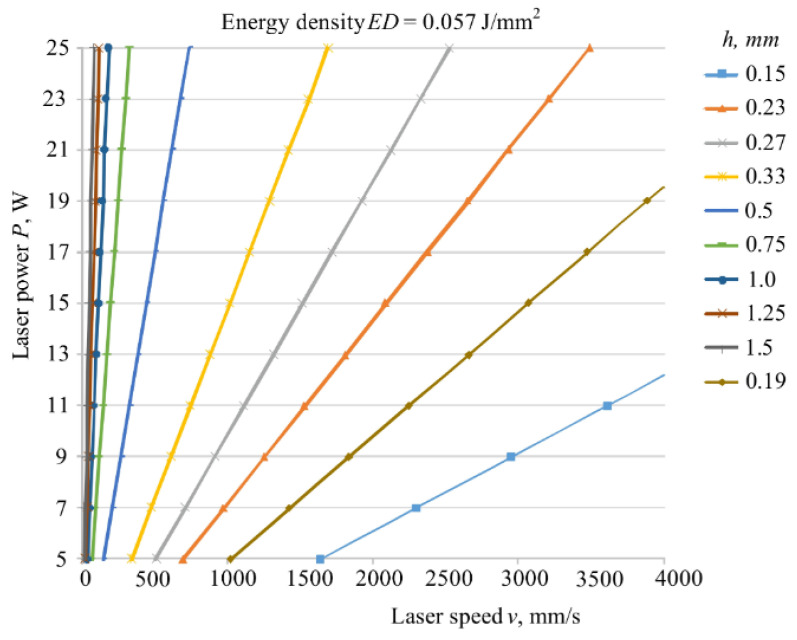
Diagram for selection of SLS process parameters [151].

The results of an experimental investigation on the influence of the energy density on the surface and cross-section porosity of components made from twice-recycled polyamide PA2200 are given in [148]. SEM analyses revealed that the SLS samples built with medium energy density had the lowest porosity both on the surface and in the cross section. According to Madžarević et al. [149], the energy density is also a critical process parameter in the SLS printing of biopolymers for solid dosage forms (tablet production) because it affects the physical, mechanical, and morphological characteristics of the tablets. The effects of the process parameters (laser power, scan speed, hatch spacing, and scan length) on mechanical properties (strength, modulus of elasticity, and elongation) of PA12 samples as well as economic aspects of the SLA process can be found in [150]. It was found that the mechanical properties are most affected by the hatch spacing. The laser power and scan speed are identified as significant process parameters, while the scan length had only a minor effect on the modulus of elasticity and no influence on the part strength and elongation. In the paper by Pilipović et al. [151], the mathematical model for energy density (Equation (1)) was modified and expanded by the overlay ratio *x = d/H* to include laser beam diameter *d*, which is found to be an important parameter in terms of mechanical properties and the production time. Using the new mathematical model, the values of SLS process parameters (energy density, laser power, scan speed, hatch distance, and layer thickness) were calculated to provide the best tensile properties of PA12 specimens. In this regard, a diagram for quick selection of the optimal combination of the parameters was created (Figure 20).

**Figure 21:**
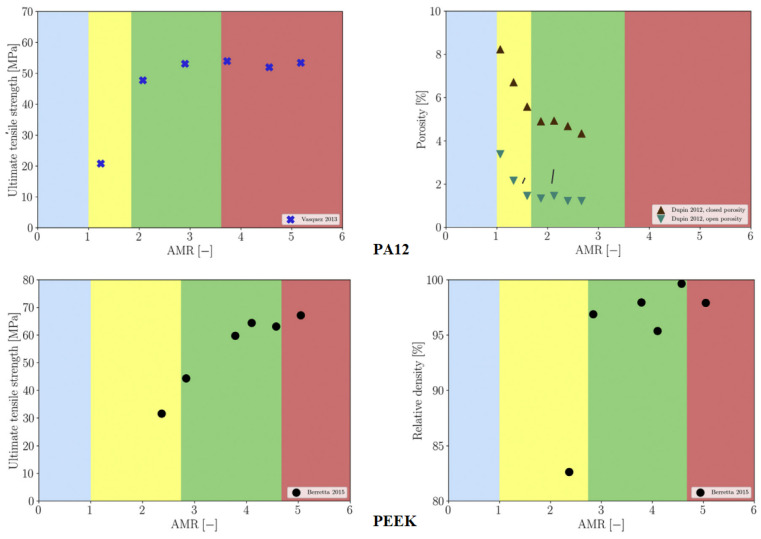
Laser-sintered PA12 and PEEK part properties as functions of the attenuation melt ratio (AMR) with color-coded process regimes (light blue: no melting; yellow: pool too shallow; green: process window; red: degradation) [152].

Bierwisch et al. [152] constructed normalized process diagrams that relate material properties and process parameters for processing PA and PEEK. Each diagram describes process windows for optimal SLS part properties and can thus be used to identify suitable process parameters for a given material (Figure 21). Chung et al. [153] used design of experiments (DOE) to optimize the process parameters for fabricating tissue engineering scaffolds from PCL composites reinforced with different volume fractions (10–30%) of tricalcium phosphate (TCP). In their study, Partee et al. [154] determined the optimal SLS parameters for processing both solid and porous PCL scaffolds using a systematic factorial design of experiments. Several test scaffolds were manufactured according to the optimal parameters with a dimensional accuracy of within 3–8% of design specifications and a density of approximately 94% relative to full density. Drummer et al. [142] performed a series of thermo-analytical and mechanical tests to identify the building parameters for processing various polymer materials (PA12, POM, PE-HD, PP, and PEEK HP-3) that enable powder fusing (i.e., complete layer bonding) while keeping energy density as low as possible to prevent material degradation. To improve the flowability, POM powder was mixed with 0.2 wt % of Aerosil. Similarly, 0.4 wt % of carbon black was added to PE-HD powder to limit the penetration depth of the laser beam. Wu et al. [136] experimentally verified that transparent and white polymers (HDPE, PMMA, and PLA) with an average particle size of 60 μm could be processed by SLS (melted and subsequently sintered together) using 2 μm lasers without any additives.

## 5. Influence of the Structure and/or Inner Structure on Part Properties

Additive manufacturing is a layer-based, automated manufacturing process in which 3D parts are produced layer by layer directly from a 3D CAD model. The mechanical properties of 3D printed objects are influenced not only by several process parameters but also by the structure, structure density, and orientation of the applied grids and layers. 

Thus far, research has mainly been in the direction of investigating the influence of structure on the mechanical properties of PLA samples. Torres et al. [155] performed torsion tests on PLA samples with different layer thicknesses, infill densities, and heat treatment after processing. They found that the layer thickness, infill density, and infill direction have a great influence on the strength and that the infill density and the heat treatment affect the ductility of the specimens. Different infill densities and infill directions (in the direction of tensile loading and perpendicular to tensile loading) lead to different results in the tensile test. The tensile strength was 32 MPa in the best case and 5 MPa in the worst case.

**Figure 22:**
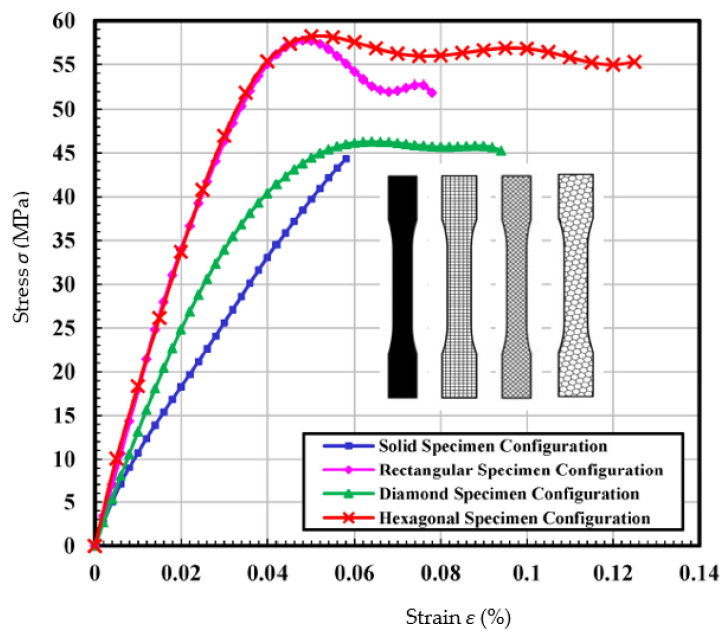
Average engineering stress–strain curves for solid, rectangular, diamond, and hexagonal infill test specimen configurations [157].

The effects of infill (shape and infill density) on the mechanical properties (tensile and flexural strength, tensile modulus) of PLA specimens can also be found in [156,157,158,159]. Cerda-Avila et al. showed that the FDM process parameters that have the greatest influence on the mechanical properties of the parts are the filling density and the printing direction. The modulus of elasticity and tensile strength are proportional to the filling fraction, but the strain is independent of the process parameters and depends on the conditions of the unprocessed material. No significant difference in the values of structural properties was found between the flat and on-edge manufacturing orientations; however, these values were much lower for the upright fabrication orientation [156]. Harpool et al. [157] investigated the relationship between the geometric shapes of the infills on the 3D printed PLA plastic and the tensile properties. The stress–strain curves showed brittle behavior for the solid-filled specimens with the lowest recorded modulus value and percent total strain. Failure occurred suddenly and without warning under the tensile loads. All the non-solid-filled specimens exhibited relatively higher yield stress, Young’s modulus, and ultimate stress values compared to the solid-filled specimens. In particular, the hexagonal configuration exhibited the highest values for modulus and tensile stress, while the values for the other properties were reasonable due to structural strength (Figure 22). A difference of ~2% was observed between the experimental and simulated results of the specimens with solid and hexagonal infill. The specimen with hexagonal infill proved to be superior in terms of tensile strength [157].

Aloyaydi et al. [158] also investigated the effects of different infill patterns (triangle, lattice, quarter cube, and tri-hexagon) on the mechanical response of 3D printed PLA samples. In this study, the low-velocity impact test, energy–time and force–displacement, and compression response were investigated and presented. The results showed that the triangular pattern produced the highest absorbed energy in the LVI test because more layers sheared/contacted perpendicular to the tool (hemispherical insert), while the grid pattern exhibited the highest compressive strength because more layers were aligned along the compressive loading direction. The SEM fracture surface pattern of the triangular infill pattern resulted in effective bonding of the lattice and layers, a lower number of voids, a greater number of circular strand marks, and the absence of lattice lines, leading to improved mechanical properties [158]. Akhoundi et al. [159] investigated concentric, rectilinear, Hilbert curve, and honeycomb infill patterns with different degrees of filling and their influence on the mechanical properties of PLA parts. The concentric pattern resulted in the highest mechanical properties (tensile and flexural strength), which can be attributed to the alignment of the grids with the loading direction. Although the higher infill levels resulted in higher values for tensile and flexural strength and modulus, this is not as obvious when weight is considered. The results show that the specific tensile and flexural moduli are highest at the lowest filling percentages. The specimens printed with the Hilbert curve exhibited poorer properties at the low filling percentages (20% and 50% in this case). However, at a fill level of 100%, a dramatic increase in tensile and flexural strength was observed, making them superior to the rectilinear and honeycomb patterns and equivalent to the concentric patterns. The honeycomb pattern proved to be the least successful at a fill level of 100%, although it exhibited better properties at a low fill level. The researchers also studied the morphology of the broken surfaces using SEM. The microscopic images show that only a small number of tiny voids are present in both the concentric and Hilbert curve patterns, justifying the higher mechanical properties of the printed samples with these patterns at the highest filling level. The presence of large voids in the printed specimens with the honeycomb pattern is the reason for their lowest strengths at this filling level [159].

**Figure 23:**
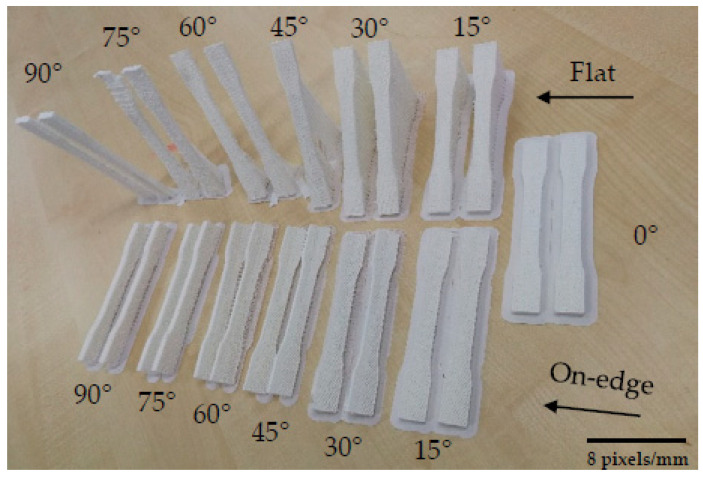
Three-dimensional printed PLA samples with different build orientations [160].

Apart from the filling density and patterns, there are numerous studies on the influence of the printing deposition direction and build-up on the mechanical properties of biopolymers [160,161]. These influences have been investigated by Dezaki et al. from different build-up directions in two directions: flat and at the edge (Figure 23).

**Figure 24:**
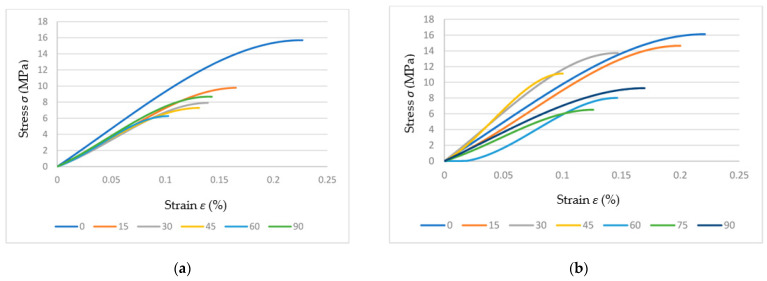
Stress–strain curves for (**a**) flat samples and (**b**) on-edge samples [160].

They also analyzed the strength of different infill patterns (such as honeycomb, rectilinear, lattice, cubic, Hilbert curve, and others) using finite element analysis (FEA). The specimens printed at the edge had better mechanical properties compared to the flat specimens. In both directions, the 0° specimen had the highest strength (Figure 24) and the best quality compared to the vertical and perpendicular specimens.

FEA results showed that honeycomb and lattice patterns were the strongest among the other patterns, while they had lower weight [160]. Martin et al. [161] investigated the influences of the printing deposition on the tensile properties of PLA samples. The yield modulus values obtained in the tensile test showed a decrease of 15.96% with concentric deposition and 27.07% with zigzag deposition compared to the manufacturer’s data [161].

Another interesting area of research is the investigation of the ability of various 3D printed infill structures to recover after unloading. These types of porous structures can be used for personal protective equipment, aerospace structures, or medical bone implants due to the shape-memory properties of the material. The response of the material after a defined quasi-static load and the ability to recover the original 3D printed shape were investigated. Ehrmann et al. [162,163] investigated the recovery ability of porous PLA cubes. In their work, 3D honeycomb and 3D gyroid infill structures were tested. The honeycomb structure exhibited significantly higher resilience (higher loads for the same impact) because the honeycomb specimens have vertical walls that can fully counteract the applied loads. The gyroid cube specimens exhibited better recovery. In their next work, the same authors investigated the influence of the orientation of the applied pressure on the recovery properties of the gyroid cubes with different infill contents. They found that for the applied gyroid pattern, indentation on the side parallel to the layers gives the worst recovery, since the layers are almost completely separated, while indentation on the side perpendicular to the layers or diagonally gives much better results.

**Figure 25:**
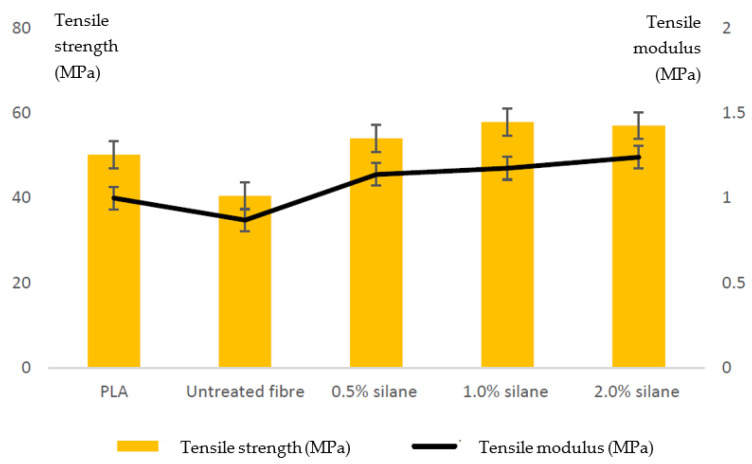
Results of composite tensile tests [164].

Natural fiber-reinforced polymers, such as acrylonitrile butadiene styrene (ABS) and biopolymers (e.g., PLA), have been the subject of research by many authors. The main problem with natural fiber-reinforced polymers is that the natural fiber is hydrophilic while the polymer matrix is hydrophobic. This problem could be overcome by modifying the surface of the natural fibers through a chemical treatment with a combination of an alkaline treatment and a silane coupling agent [164]. Jamadi et al. [164] evaluated the effects of alkali and silane treatment on the mechanical properties of 3D printed PLA samples reinforced with natural kenaf fibers. The composite filament of PLA and kenaf was prepared using a twin-screw extruder. Untreated natural fiber composites exhibit the lowest strength because, unlike in treated composites, stress cannot be distributed uniformly over the surface and interfacial adhesion is poor. In summary, for natural fiber modifying composites, the optimum silane concentration for surface treatment of natural fibers is the most important element to be considered, as this experiment also proves that the application of a higher silane concentration can lead to fiber damage. For example, the graph in Figure 25 shows that samples with a silane concentration of 2.0% had lower strength than samples with a concentration of 1.0% [164].

The next very common method of reinforcing PLA with natural materials is the production of PLA–wood composites. Cuan-Urquizo et al. [165] studied the effects of different infill configurations on the mechanical properties of PLA–wood composites. PLA–wood specimens were fabricated with two different infill topologies: hexagonal and star-shaped with different filling ratios (20%, 30%, 40%, and 50%). All PLA samples were fabricated without outer walls. The results showed that the infill density of the filling had a significant effect on the effective mechanical properties. The topology also had an influence on the mechanical properties. The effective stiffness of the hexagonal topology was 60% to 100% higher than that of the star topology at low filling percentages. However, at higher infill fractions, the star-shaped configuration was 21% to 78% stiffer than the hexagonal topology [165]. Ayrilmis et al. [166] examined the effects of gyroid structure on the mechanical properties (flexural and compressive strength and Brinell hardness) of PLA wood panels with different sizes and face layer dimensions, as well as without face layers. The results show that the addition of the top and bottom face layers significantly increases the flexural and compressive strength. The flexural strength and modulus of the specimens are significantly improved with an increase in the thickness of the face layers. The compressive strength (parallel to the edge) of the rectangular specimens showed a similar trend to the flexural properties. As a conclusion of the study, the authors recommend panels with gyroid structures with an outer layer of at least 2 mm to achieve good flexural and compressive properties and panel hardness.

**Figure 26:**
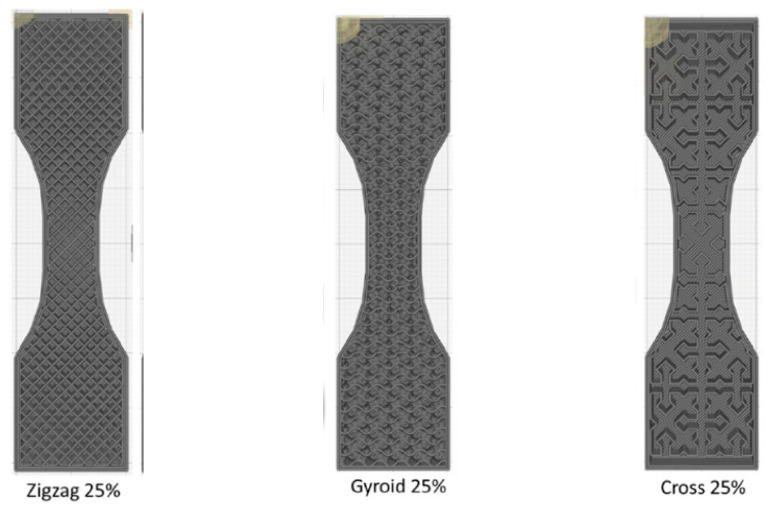
Three different tensile specimen patterns (zigzag, gyro, and cross) with 25% infill density [167].

Besides wood, PLA composites are often reinforced with carbon particles and fibers. There are two possible production principles for polymer composites with additive technologies. The first is to produce composites and products in one step, printing a polymer matrix and adding different types of fibers. The second way is much simpler and essentially involves printing the finished composite material, such as polymer–carbon filaments. Carbon fiber filaments use tiny fibers incorporated into a base material to improve the properties of that material. There are several common filaments with carbon fiber filling, including PLA and polyethylene terephthalate glycol (PETG). Guessasma et al. [167] investigated the use of different infill patterns to improve the tensile properties of printed carbon PLA structures. The samples were fabricated from PLA filaments reinforced with 10 wt % ground carbon fibers with a maximum particle size of 0.15 mm. In the work, three types of infill patterns (cross, gyro, and zigzag patterns), shown in Figure 26, were combined without top layers and with four filling density degrees (25%, 50%, 75%, 100%).

**Figure 27:**
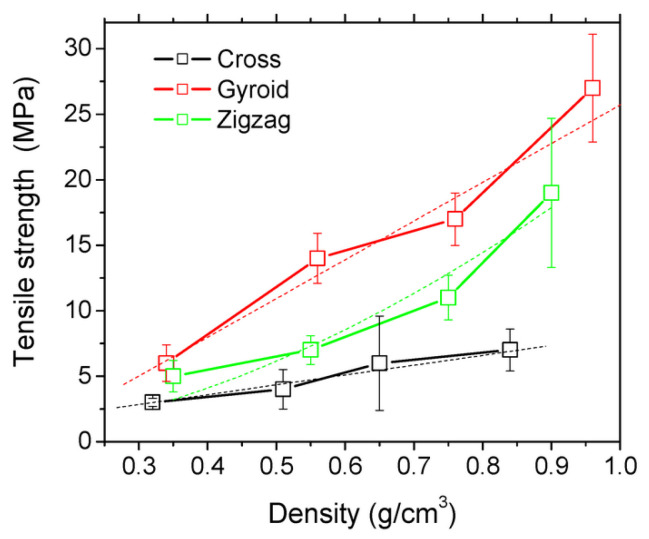
Correlations between engineering constants and the density of PLA–carbon fiber patterns with different infill densities ranging from 25% (point with lowest density) up to 100% at points with the highest material density [167].

The results in Figure 27 show that the infill density affects the mechanical properties, although these dependencies vary for the different filling patterns due to the different compactness of the patterns [167]. The gyroid pattern is the best option for improving the mechanical strength, while zigzag and cross patterns are more suitable for promoting large strains, especially at low infill rates.

In contrast, Saleh et al. [168] investigated three cell topologies (i.e., diamond, gyroid, and primitive) on the compressive properties of pure PLA and 3D printed PLA–carbon composites with 15% carbon fibers in the PLA matrix. The results show a change in compressive strength and modulus due to the influence of CF incorporation, cell type, and size. All structures showed a higher compressive modulus for PLA–carbon structures compared to the neat PLA material. However, the compressive strength of the primitive and gyroid structures was higher than that of the pure PLA samples.

Besides PLA, the most commonly used bio-based polymer in additive manufacturing, there are other materials such as bio-polyethylene (BioPE), polyhydroxyalkanoate PHA, PLGA, and PCL. These materials are generally not as well researched as PLA. In the review of the available literature, no papers were found that analyzed the various structures and, in particular, the effects of structure on mechanical and other properties. Most of the work on these materials examines the effect of various modifiers and reinforcements on improving adhesion between layers compared to the neat material.

One of the biggest problems with 3D printing from BioPE is the large volume change during cooling, which can lead to the warping of parts and weak bonds between two layers. One way to improve the dimensional accuracy and the interaction between the welded layers of the printed parts is to use inorganic fillers, such as pozzolan. Schiavone et al. [169] investigated the influence of structure and pozzolan content on the mechanical properties of BioPE composites. Pozzolan improved the interlayer welding and significantly reduced the horizontal voids in the BioPE composite structure. The pure BioPE specimens and the composite specimens with a pozzolan content of 20% showed no significant differences. However, by increasing the filler content, higher stiffness was achieved. These results are similar to those of previous studies with this material. In addition, Tarres et al. significantly improved the number of voids between the compression layers in the structure by adding thermomechanical pulp fibers [169,170]. Tian et al. [171] studied polyhydroxyalkanoate–wood flour composites without any additives. Compared with polyester PLA, PHA has a shorter development history, but its development potential and application range are greater. PHA has relatively high crystallinity and is similar to conventional polymers such as PE and PP. The authors conclude that wood flour improves the printability of PHA. The melting temperature and crystallization temperature of the wood flour (WF)/PHA composites were much higher than those of neat PHA. Higher content of WF decreased tensile and impact strength and significantly increased the tensile and flexural moduli. The authors also investigated the effect of raster angles (0°, 45°, and 90°) on the mechanical properties. The specimen with a grid angle of 90°/90° exhibited lower ultimate stress and strain compared to specimens with grid angles of 45°/45° and 0°/0° [171].

## 6. Complexity of Additive Manufactured Parts

**Figure 28:**
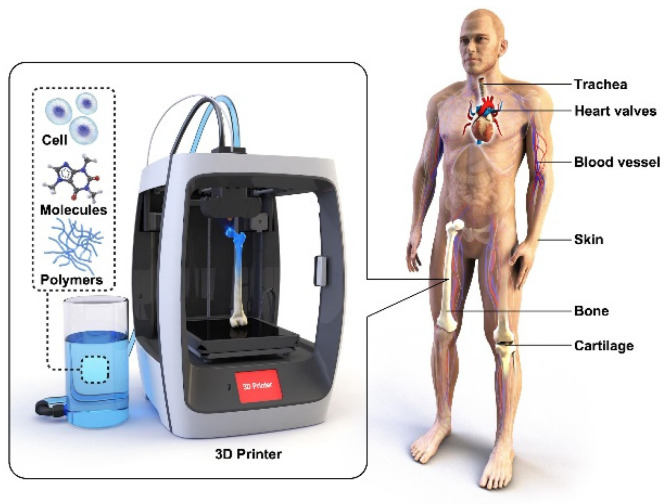
Various scaffolds for tissue engineering obtained by AM technologies [173].

AM technologies allow for the design and fabrication of parts and devices with complex geometry, complex (multi)material compositions and designed property gradients, and highly sophisticated multiscale structures (from nano- to macrostructures). As previously mentioned, practically any shape that can be constructed in a 3D CAD program can be produced using AM technologies. AM-manufactured parts from biopolymer materials differ greatly in terms of shape, size, internal structure, amount of details/resolution, and other design aspects. The most complex forms/configurations are encountered in the medical and biomedical fields, where AM technologies have opened new possibilities for manufacturing complex, highly customized components such as prostheses and orthoses, dental and medical implants, joint replacements, dentures, dental crowns and bridges, and splints. Within the medical field, AM is also widely utilized to fabricate a variety of medical devices, surgical equipment, instruments and training models, and drug systems. In addition to single-component manufacturing, additive manufacturing (AM) is used to create multimaterial and multipart structures that are difficult or impossible to manufacture using conventional manufacturing techniques [172]. Such structurally or chemically gradient components have the potential to operate as multifunctional biomedical devices [109]. Medical scaffolds are one of the most sophisticated and complex structures created by AM from biopolymers. Scaffolds are used in both soft and hard tissue engineering as support structures to facilitate cellular growth and proliferation upon implantation into the patient [173]. Figure 28 shows different types of 3D printed scaffolds for regenerative medicine. 

**Figure 29:**
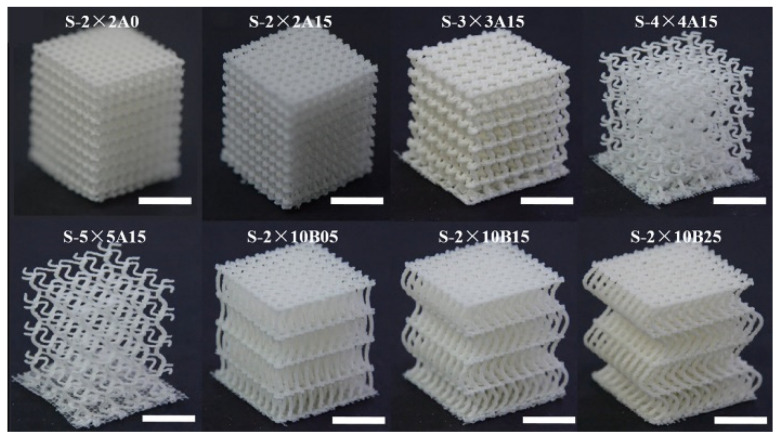
Geometry of SLS-fabricated PCL scaffolds [174] (scale bar: 10 mm).

A medical scaffold, in addition to having favorable biological and mechanical properties, should have a porous structure with adequate interconnected pore networks and pore size for effective cell transport and growth [114]. Therefore, scaffold design (material and geometry optimization) and, specifically, AM process design (selection of process parameters) are extremely challenging issues. As an illustration of the above, Figure 29 shows the SLS-fabricated scaffolds with different sinusoidal architectures (periods and amplitudes) and the diameter of filaments being in the range of 701.3 ± 113.8 μm [174]. By adjusting the amplitude-to-period ratio of sinusoidal filament, the elastic modulus and mechanical properties of the scaffold could be fine-tuned in line with the required function for soft tissue engineering. 

**Figure 30:**
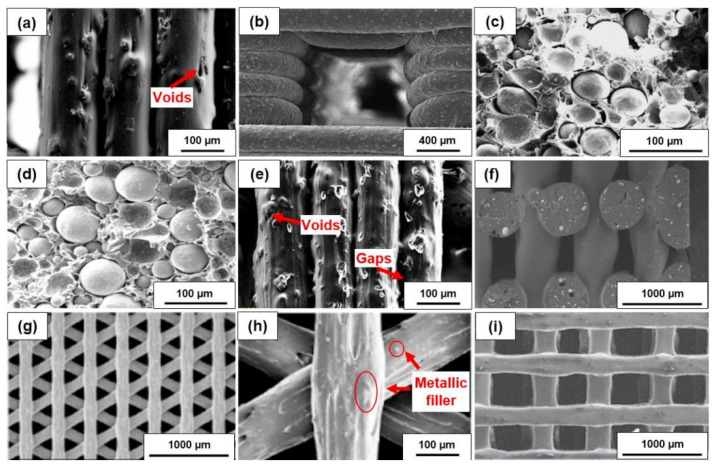
Scanning electron microscope (SEM) images of the structure of different polymer/metal composite scaffolds obtained by FDM: (**a**) PLA/10 vol% 316L stainless steel; (**b**) PLA/15 vol% Ti; (**c**) cross section of the PLA/24 vol% Cu; (**d**) cross section of PLA/30 vol% bronze; (**e**) PLA/10 vol% Fe, (**f**) PLA/7 vol% Mg, (**g**,**h**) PCL/6.8 vol% Mg; and (**i**) PCL/6.1 vol% Mg [180].

Growing demand for scaffolds with improved functional performance has led to the development of multimaterial scaffolds. Compared to single-material scaffolds, multimaterial scaffolds are more sophisticated structures since their framework consists of different materials joined together. Thanks to the unique capabilities of AM technologies to build objects layer by layer, each material component can be positioned accurately, allowing for the scaffold properties to be tailored by varying the percentage and architecture of material components. Feng et al. [175] mixed biodegradable poly(l-lactide) (PLLA) powder with PEEK and *β*-TCP powders to fabricate three-phase scaffolds via SLS for bone regeneration. The scaffolds had 3D porous structures with dimensions of Ø 15 mm and a height of 21 mm as the pore sizes and strut sizes were approximately 450 and 500 µm, respectively [175]. Several AM techniques, including the two-photon polymerization process, enable the fabrication of 3D composite/hybrid scaffolds with pores of sub-micrometer size and a porosity of over 80%, as reported in [176]. Very often, biopolymers are combined with other types of materials (metals, ceramics) to improve the function and biological and mechanical properties of scaffolds. A composite scaffold structure that incorporates metallic or bio-ceramics fillers into a polymer matrix has been shown to be beneficial in terms of bioactivity, mechanical properties, and control of porosity and degradation rate [177,178,179,180,181]. The composition, geometry, and particle distribution of several polymer/metal composite scaffolds obtained by FDM are depicted in Figure 30. Furthermore, various polymer/metal blends such as PLLA/Mg [182] and polymer/bio-ceramic blends such as PEEK/HA (hydroxyapatite) [183], PCL/HA, PLLA/HA, and PLGA/HA [184] are used in the fabrication of composite scaffolds via SLS technology.

**Figure 31:**
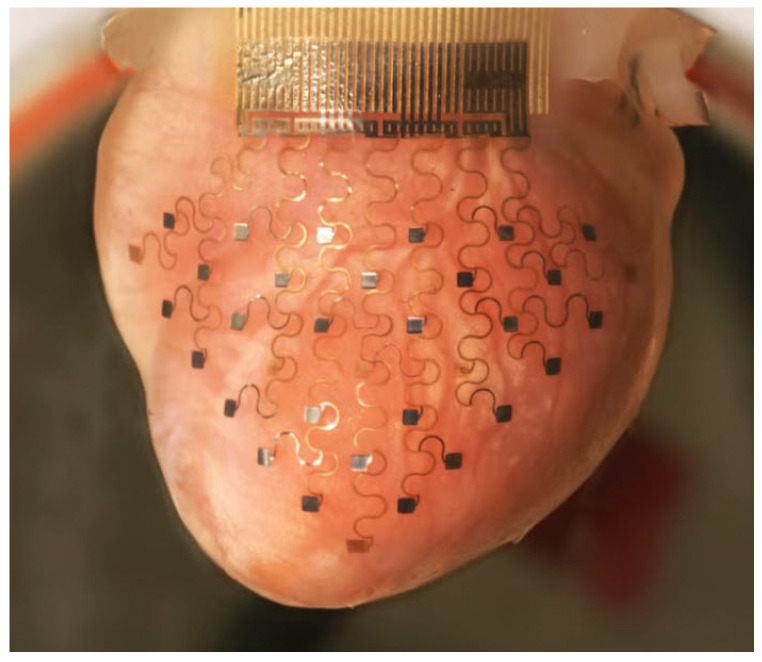
Cardiac membrane fitted to a rabbit’s heart [186].

Scaffolds are not the only medical components and biomedical devices with intricate geometries and multimaterial structures. In the paper by Zarek et al. [185], conductive materials and PCL biopolymer are combined with a modified SLA, inkjet-based printer to create shape-memory objects that may be used in soft robotics, minimally invasive medical devices, and sensors. A cardiac sleeve/membrane, intended for replacing pacemakers, is an example of an AM-fabricated biomedical device with sophisticated multimaterial architecture and complex geometry [186]. As shown in Figure 31, the cardiac sleeve comprises a thin, stretchable membrane (made of biocompatible silicone) that bears an imprinted spider web-like network of sensors and electrodes. This membrane is custom-designed to fit over the heart and contract and expand with it as it beats. Zarek et al. [187] have developed a smart tracheal–bronchial stent with complex geometry and shape-memory function that is capable of changing shape in response to changes in the environment, such as airway growth. Zhu et al. [188] recently reported on a hydrogel-based strain sensor printed directly on a porcine lung under respiration-induced deformation. As the ultimate personalized medical components, patient-specific biosensors must be compatible with nonplanar organ structures and tissue surfaces with complex geometrical features.

**Figure 32:**
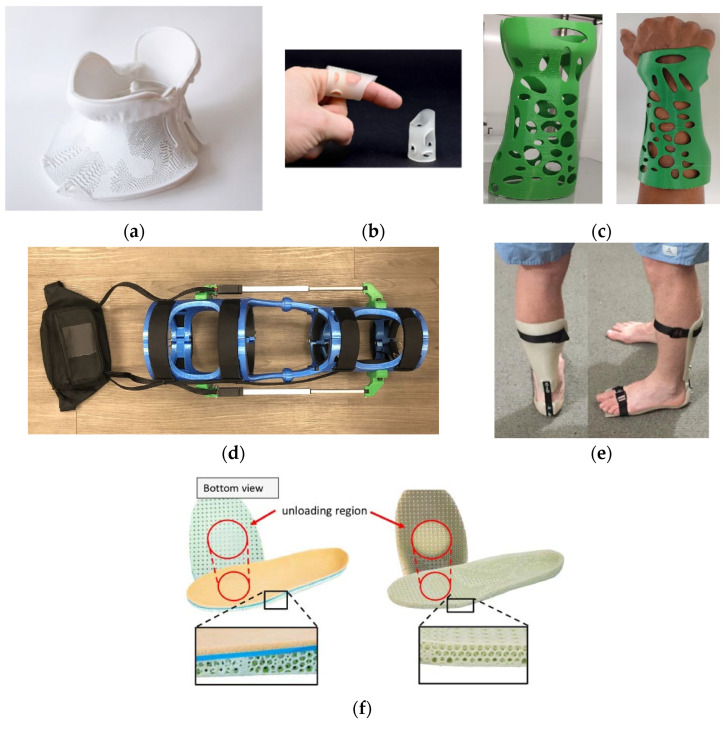
Orthosis devices for (**a**) neck [193], (**b**) finger [189], (**c**) hand [190], (**d**) knee [191], (**e**) ankle [194], and (**f**) foot [192].

The quality of the rehabilitation process can be improved and accelerated using an additive manufacturing process in a way to produce personalized products for each patient, which is more advantageous than prefabricated orthoses. Different orthoses can be fabricated using FDM, SLS, or SLA technology from many polymer materials, such as PLA, ABS, PETG, and nylon. In combination with the digital scanning technique, additive technologies are a powerful tool in the rehabilitation process; in a short time, with little effort, 3D scanned models of extremities can be created without any discomfort for the patient. Most orthoses are parts with complex shapes, and for this reason, it is almost impossible and very expensive to personalize them for each patient in a conventional production way [189]. In Figure 32, different complex 3D printed orthoses devices, for the lower and upper extremities, are presented [189,190,191,192,193,194].

Additive technologies have been used for indirect implant production; molds for materials that were not biocompatible were produced (e.g., plaster), sterilized, and used for implant forming [195,196]. Regardless of the indirect way of production, complex implants could be produced, and 3D printers made surgical interventions much easier. 

**Figure 33:**
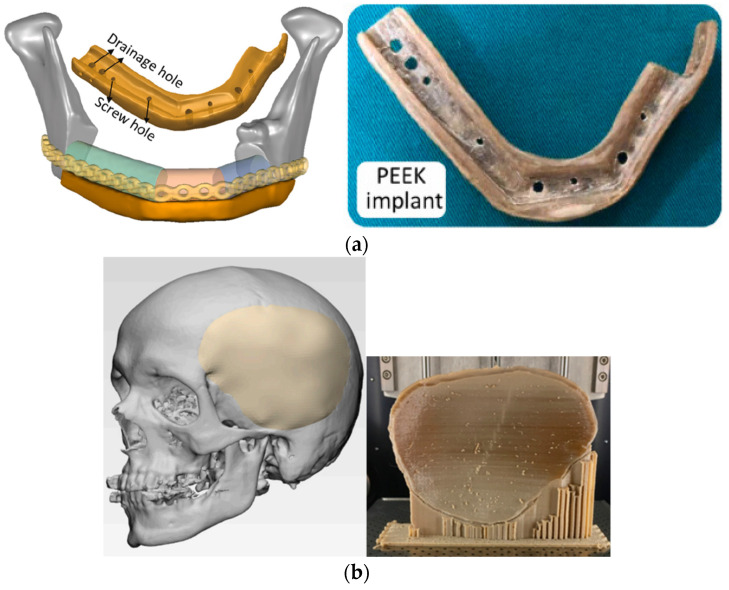
Two types of PEEK implants: (**a**) mandible implants 197 and (**b**) cranial bone implants 131.

With new material development, additive technologies find an important place in bone reconstruction, i.e., implant production. Today, one of the most used materials for implants is PEEK, which has excellent mechanical properties and heat resistance and at the same time is easily printable. According to these characteristics, it can be used not only for statically loaded implants but also for dynamically loaded implants, such as mandibles. For that purpose, Kang et al. [197] used PEEK in combination with a titanium plate for 3D printed mandible reconstruction (Figure 33a). Previously impossible-to-produce shapes, such as customized cranial implants, now with additive technology represent no challenge for engineers and surgeons. In [131,198], authors provided a reconstruction of cranial bone and replaced damaged parts of bones with PEEK implants (Figure 33b).

## 7. Conclusions

Nowadays, manufacturing with additive technologies is often used to produce complex parts for prototyping, individual production, and small-batch production. AM has been applied in various fields in recent decades, from aerospace, automotive, electronics, and civil engineering to food processing, art, apparel, and medicine. Since the field of AM is too extensive to be considered in general, the authors have focused on the applications of biopolymers in this paper. To assess the scientific impact of particular AM technologies applied to biopolymers, an analysis of the WOS database was performed. It can be seen that the major AM technologies (i.e., SLS, SLA, FDM, etc.) deliver huge numbers of papers. For example, the keyword search of “additive manufacturing”, “3D printing”, and “polymers” delivered 9264 hits in WOS, of which 7739 were published in the previous five years. Further focusing on the field of biopolymers delivered 91 results from 2018 onward, most of which are related to the various applications in the medical field. Research has shown that biopolymers are used for various applications, and in particular, the most commonly utilized biopolymer, PLA, is widely used in the production of consumer parts, demonstration and presentation parts, and precise applications where medicine plays a crucial role. In addition, most of the complex and advanced materials, such as PGA, PCL, PLCL, PEEK, and PEG, which have been researched in recent years, are used in medicine. These materials are often used in tissue engineering and replacements of damaged human tissues. With their biocompatibility and later biodegradable properties, they offer optimal support for tissue regeneration. In this area, PCL is used in 29% of all applications while PLA and PGA are used in 11% of all applications. In addition to medical applications, due to its low melting point, PLA is also widely used in common engineering applications mainly used for presentation purposes. In contrast, parts with lower strength are also produced from PLA since its tensile strength is only 50 MPa, and the impact strength measured according to the IZOD specimen reaches 96 J/m.

In the introduction, the extent of research on biopolymer materials is presented, starting from 1984, when printing of these materials was invented, and extending to complex research on bioprinting of skin and heart valves in the previous five years. For these materials, the technologies of inkjet bioprinting, selective laser sintering, stereolithography, fused deposition modeling, extrusion-based bioprinting, and scaffold-free printing (the so-called Kenzan process) are crucial for complex medical applications ranging from tissue preparation to various prosthetic devices, specialized surgical equipment, instruments, training models, and drug delivery systems. Single-material and multimaterial AM technologies are used in these applications. The research performed is focused on the increasingly complex production of medical equipment and parts for tissue replacements and building entirely artificial human organs made mainly from biopolymers. 

The influences of technological parameters on the quality of manufactured parts are presented, for which particular technological parameters, such as the layer thickness, build-up rate, modifications of the material properties through the AM process itself, surface roughness of the produced parts, environmental impact of the AM process, selected infill density, and surface layer thickness, influence the mechanical, optical, and thermo-physical properties of the produced parts. 

In the case of the FDM process, the layer thickness and printing/scan speed have a greater influence on the quality and mechanical properties of the produced parts than other parameters such as printing head temperature and printing bed temperature. The density of the filling and the shape of the filling are crucial in all AM technologies where larger parts are to be produced. However, in the case of FDM, these two parameters have a significant impact on the mechanical properties of the manufactured parts but only a minor influence on the surface quality and morphological changes of the produced part. 

Similar problems can be observed in SLA and SLS technologies; both technologies are dependent on the energy source necessary to bind the material into the solid part. The main difference between both technologies is that SLA uses a liquid material that needs to be cured into a solid part, while SLS uses a material that consists of small particles, and laser power is used to add the necessary energy to remelt it. 

The research on AM of several biopolymer parts produced by SLA technology showed that five major properties and technological parameters of the parts need to be analyzed: the tensile strength, building time, part roughness, part accuracy, and post-curing time. The last item is mostly indispensable to achieving the required mechanical properties of the SLA-produced part. The post-curing time is mainly influenced by the SLA process itself where the technological parameters affect the material curing and layer bonding. Furthermore, with proper selection of the polymer curing and later post-curing, the residual stresses can be decreased, resulting in the minimization of distortion, warpage, and creep.

The crucial influential technological parameters of SLS technology are mostly connected with the energy source implemented: the laser beam. This influences the melting of the particles, where their size, distribution, and shape are of the highest importance. However, not only the particles’ sizes and shapes but also the material’s thermo-physical properties with the heat capacity and melting temperature are of the highest importance. The environment in this technology influences the process itself, which is similar to the case of FDM. However, in the case of SLS, the powder feeder and powder bed temperatures must be controlled, and the temperature and humidity of the environment are also not to be neglected.

Several studies have also observed the influence of the positioning of the printed part during the AM on its mechanical properties, which are critical to the quality of part production. The positioning of the part in the working area of the AM machine is crucial in all presented technologies and is particularly important in the case of tensile loading of the produced parts which must be, if possible, selected perpendicularly to the build-up direction. 

Finally, the AM parts can be produced with full density or with infill densities as low as 10% of the full volume only. In this case, the strength of such parts can be significantly lower in comparison to the parts with full density, but their production times are significantly shorter, and their mass is also reduced. This is especially important when large parts are produced, which is important in the production of medical prosthetic parts. Furthermore, the mechanical properties of parts with less than 100% infill are influenced by not only the infill density but also the infill pattern. Considering these parts, the lower strength can be improved with a better material or the implementation of biopolymer composites. 

Focusing on the production of smaller parts in the medical sector, the proper combination of infill density and infill structure can decrease the mass of the parts substantially without a drastic decrease in their strength. In contrast, the elastic stiffness of such parts can be up to 60% larger in the case of proper selection of infill density and infill structure. Several papers analyze the technological parameters of the FDM process, but there is still a lack of their optimization, especially when the process optimization during the build-up of the produced part itself is considered. 

It is also clear from the review that the main future applications of biopolymers are the implementation of fabrics, composite structures, and biopolymer nanocomposites and the spread of 4D. The so-called 4D printing technologies will enable the implementation of smart printing concepts as well as smart time-dependent materials that are able to react to temperature, magnetic fields, electric fields, cumulative cyclic strains, etc. Using these technologies, sensors that can report their state after implantation into the human body could be inserted into artificial biopolymer tissues or even human organs. Targeted modifications of material properties through the creation of biopolymer composites as well as nanocomposites for innovative medical and engineering applications will have a significant influence on the creation of such printing technologies and dedicated materials for 4D applications.

## Figures and Tables

**Table 1:** Number of scientific works found in Web of Science.

Keywords	Number of Scientific Works
“additive manufacturing” or “AM” and “3D printing”	69,341
stereolithography (SLA)	17,699
selective laser sintering (SLS)	17,366
direct metal laser sintering (DMLS)	1123
electron-beam melting (EBM)	8538
selective heat sintering (SHS)	16,220
selective laser melting (SLM)	16,474
fused deposition modelling (FDM) + fused filament fabrication (FFF)	16,645
laminated object manufacturing (LOM)	99
laser-engineered net shaping	232
Summary	147,263

**Table 2:** Influence of main printing parameters on part properties.

Printing Parameter	Mechanical Properties	Surface Quality	Morphological Changes
Layer thickness	+	+	−
Printing head temperature	+	−	+
Printing bed temperature	+	−	+
Printing speed	+	+	−
Infill percent	+	−	−
Infill shape	+	−	−

## Data Availability

Not applicable.

## Abbreviations

2PP	two-photon polymerization
3D	3-dimensional
4D	4-dimensional
ABS	acrylonitrile butadiene styrene
AM	additive manufacturing
AMR	attenuation melt ratio
ANOVA	analysis of variance
ASTM	American Society for Testing and Materials
BioPE	bio-based polyethylene
CAD	computer-aided design
CF	carbon fiber
Cu	copper
DLP	digital light processing
DMLS	direct metal laser sintering
DOE	design of experiments
EBB	extrusion-based bioprinting
EBM	electron-beam melting
FDM	fused deposition modeling
Fe	ferrum
FEA	finite element analysis
FFF	fused filament fabrication
HA	hydroxyapatite
HDPE	high-density polyethylene
LAP	lithium phenyl-2,4,6-trimethylbenzoylphosphinate
LENS	laser-engineered net shaping
LOM	laminated object manufacturing
LVI	low-velocity impact
Mg	magnesium
PA12	polyamide 12
PA6.10	polyamide 6.10
PA66	polyamide 66
PA2200	polyamide 2200
PCL	polycaprolactone
PDA	biopolymer polydopamine
PE	polyethylene
PEG	polyethylene glycol
PEGDA	polyethylene glycol diacrylate
PEEK	polyether ether ketone
PETG	polyethylene terephthalate glycol
PGA	polyglycolic acid
PHA	polyhydroxyalkanoate
PLA	polylactic acid
PLCL	poly(L-lactide-co-e-caprolactone)
PLGA	poly(lactide-co-glycolide)
PLLA	poly(l-lactide)
PMMA	poly(methyl methacrylate)
POM	polyoxymethylene
PP	polypropylene
PVA	polyvinyl alcohol
SEM	scanning electron microscope
SHS	selective heat sintering
SLA	stereolithography
SLM	selective laser melting
SLS	selective laser sintering
SMM	shape-memory material
SMP	shape-memory polymer
TCP	tricalcium phosphate
TE	tissue engineering
Ti	titanium
US FDA	United States Food and Drug Administration
UV	ultraviolet
WF	wood flour
WOS	Web of Science
